# Astonishing diversity—the medicinal plant markets of Bogotá, Colombia

**DOI:** 10.1186/s13002-018-0241-8

**Published:** 2018-06-20

**Authors:** Rainer W. Bussmann, Narel Y. Paniagua Zambrana, Carolina Romero, Robbie E. Hart

**Affiliations:** 1 Museo Nacional de Historia Natural, Calle Ovidio Suarez (26), Cota Cota, La Paz, Bolivia; 2 Herbario Nacional de Bolivia, Instituto de Ecología-UMSA, Campus Universitario, Cota Cota Calle 27, La Paz, Bolivia; 3 William L. Brown Center, Missouri Botanical Garden, P.O. Box 299, St. Louis, MO 63166-0299 USA

**Keywords:** Medicinal plants, Markets, Colombia, Globalization

## Abstract

**Background:**

Despite the importance of local markets as a source of medicinal plants in Colombia, comparatively little comparative research reports on the pharmacopoeiae sold. This stands in contrast to wealth of available information for other components of plant use in Colombia and other countries. The present provides a detailed inventory of the medicinal plant markets in the Bogotá metropolitan area, hypothesizing that the species composition, and medicinal applications, would differ across markets of the city.

**Methods:**

From December 2014 to February 2016, semi-structured interviews were conducted with 38 plant vendors in 24 markets in Bogotá in order to elucidate more details on plant usage and provenance.

**Results:**

In this study, we encountered 409 plant species belonging to 319 genera and 122 families. These were used for a total of 19 disease categories with 318 different applications. Both species composition and uses of species did show considerable differences across the metropolitan area—much higher in fact than we expected.

**Conclusions:**

The present study indicated a very large species and use diversity of medicinal plants in the markets of Bogotá, with profound differences even between markets in close proximity. This might be explained by the great differences in the origin of populations in Bogotá, the floristic diversity in their regions of origin, and their very distinct plant use knowledge and preferences that are transferred to the markets through customer demand. Our study clearly indicated that studies in single markets cannot give an in-depth overview on the plant supply and use in large metropolitan areas.

## Background

Like in many tropical countries, traditional knowledge in Colombia is still under-documented [1–3], despite the fact that the country could be called the “cradle of modern ethnobotany”, due to the decade long research of Richard Evans Schultes [4]. General ethnobotanical studies in Colombia have frequently focused on individual interesting species [5–13], specific medicinal applications (e.g., antiviral and antitumor activity) [14, 15], leishmaniasis [16], snake bites [17–21], crops and economically important plants [10, 22–25], or specific plant families [26–33]. Individual ethnic groups were recently treated by a variety of authors [34–38]. Columbian medicinal plant use has even been studied overseas [39].

However, in contrast to neighboring countries like Peru and Bolivia [31, 40–50], although Lima et al. [51] provided a comparison of markets in the wider Amazon.

Thus, for Colombia there is very little comparative information available about which plants are sold in Colombian markets, under which vernacular name at any given time, for which indication, which dosage information, and what kind of information about side effects vendors provide to their clients. The present study attempts to remedy this situation by providing a detailed inventory of medicinal plant markets in the Bogotá metropolitan area. We hypothesized that, on the one hand, like in Peru and Bolivia [31, 40–50] the plant and use composition of different markets would vary depending on their location and customer population, but that, on the other hand, there would overall be a large overlap in plants between markets, and larger markets would comprise an almost complete inventory of all species available.

## Methods

### Ethnobotanical inventory

From December 2014 to February 2016, semi-structured interviews were conducted with 38 plant vendors in 24 markets in Bogotá in order to elucidate more details on plant usage and provenance. The markets were chosen to cover as many neighborhoods of Bogotá as possible, thus trying to represent the complete geography of the city and its ethnically diverse population. Plants were always sold in the regular *mercados de abasto*—the regular food supply markets. Within a neighborhood, we always chose the main market, in order to make data comparable. Semi-structured interviews following [49] are under consideration of the collection standards established by [52]. During the interviews, a complete sample of all plants sold in a respective market stall was bought and all available information for each species elucidated from the vendor. Vendor participants were self-selected: every vendor in the studied markets was asked if they wanted to participate in the study, and the vendors who agreed were then interviewed. Overall about one third of the vendors in the markets participated in the interviews. Interviews were conducted only after explaining the study to all participants and obtaining their oral prior informed consent. No further ethics approval was required. All work was carried out following the International Society for Ethnobiology Code of Ethics [53] and under the framework provided by the Nagoya Protocol on Access to Genetic Resources and Fair and equitable sharing of benefits arising from their use of the Convention on Biological Diversity; the interviewed vendors retain the copyright of their traditional knowledge. Any commercial use of any of the information requires prior consensus with the informants involved, and an agreement on the distribution of benefits.

Twenty-six of the participants were women, and 12 men, with ages ranging from 25 to 70 years. The ethnicity of the vendors was not disclosed. All interviews were conducted in Spanish.

Vouchers of all species were collected, and all plant material was identified at the National Herbarium of Colombia. No material whatsoever was exported from Colombia. The nomenclature of all species follows www.tropicos.org, under APG III [54].

### Statistical analysis

Among markets we compared plant species reported as being used (unique Latin binomials, e.g., *Aloe vera*), plant uses (unique combinations of a species used for an ailment or illness, e.g., *Aloe vera* for eye irritation), and plant categories (unique combinations of a species used for a category of ailments, e.g., *Aloe vera* sensory system).

To compare the geographic/market structure of plants and plant uses, we extracted lists of unique and shared plants, plant uses, and plant categories in each market [55]. In addition to these raw counts, we also used Euclidian distance as a metric of the difference among markets.

To evaluate plant importance, we used the logarithmic informant consensus (LIC) index of [56]. Here, we considered markets as “occurrences,” so for a species:

$$ \mathrm{LICs}=\mathrm{sum}\ \Big(\mathrm{ICu}\times \ln \left(\mathrm{FCus}\right) $$

where for each use of a species ICu = (FCu − NSu)/(FCu − 1), FCu is the total reports of that use and NSu is the number of species reported for the use.

To evaluate the diversity of uses across markets, we calculated, for each plant that occurs in at least two markets, the percent of its uses that are unique to each market.

All analyses were performed using the R program package [57].

## Results

In this study, we encountered 409 plant species belonging to 319 genera and 122 families in all markets studied (Table 1). In all markets, the most common plant families used were Fabaceae (43 species, 10.5%), Asteraceae (34 species, 8.3%), Lamiaceae (24 species, 5.8%), Malvaceae (16 species, 3.9%), Solanaceae (15 species, 3.7%), and Euphorbiaceae (13 species, 3.1%). Almost all of all species were known only by their Spanish names.

**Table 1:** ᅟ

Family / Scientific name	Vernacular name	Medicinal Use categories (Ailment / Illness) *= non medicinal uses	Market
Acanthaceae			
*Aphelandra pilosa* Leonard	Cajeto	Metabolism and nutrition (High cholesterol, Obesity); Urinary system (Diuretic)	Plaza Santander
*Hygrophila tyttha* Leonard	Amansaguapos / Dominio	Nervous system and mental health (Anxiety, Tranquilizer); Non-specific symptoms and general illnesses (Seizures)	Plaza de Las Ferias; Plaza de Mercado Trinidad-Galán; Plaza de San Benito
*Justicia pectoralis* Jacq.	Amansatoros	Blood and circulatory system (Cardiac stimulant); Nervous system and mental health (Anxiety)	Plaza del 20 de Julio
*Justicia xanthostachya* Leonard	Pulmonaria	Dental health (Mouth infections); Muscular-skelettal system (Muscle relaxant); Nervous system and mental health (Sedative); Non-specific symptoms and general illnesses (Analgesic, Fever, Inflammation); Reproductive system and sexual health (Sexual potency); Respitarory system (Expectorant, Flu, Help breathing); Skin and subcutaneous tissue (Astringent, Healing)	Plaza de Las Ferias
*Trichanthera gigantea* (Bonpl.) Nees	Cajeto / Nacedero / Quiebrabarrigo / Quiebrabarriga / Cafeto	Blood and circulatory system (High blood pressure); Digestive system (Hernia, Liver problems); Infections and infestations (Syphilis, Vermifuge); Metabolism and nutrition (Obesity); Muscular-skelettal system (Rheumatism); Non-specific symptoms and general illnesses (Fever); Reproductive system and sexual health (Dysmenorrhea, Emenagogue); Skin and subcutaneous tissue (Sores); Urinary system (Diuretic, Urinary infection); Veterinary (Hernia in cows)	Plaza de Kennedy; Plaza de Las Cruces; Plaza de Las Ferias; Plaza de Mercado Trinidad-Galán; Plaza del 20 de Julio; Plaza del 7 de Agosto; Plaza del Restrepo; Plaza Samper Mendoza
Adoxaceae			
*Sambucus peruviana* Kunth	Sauco	Blood and circulatory system (Arteriosclerosis, Blood cleansing); Cultural illnesses (Purifies the body); Dental health (Mouth diseases); Digestive system (Constipation, Diarrhea); Infections and infestations (Measles); Muscular-skelettal system (Arthritis, Neuralgia, Rheumatism); Non-specific symptoms and general illnesses (Apena, Fever, Headache, Promotes sweating); Others (Sunstroke, Tonic); Respitarory system (Bronchial diseases, Bronchitis, Cough, Expectorant, Flu, Respiratory tract, Sinusitis, Throat inflamation); Sensory system (Conjunctivitis); Skin and subcutaneous tissue (Burns, Skin diseases); Urinary system (Diuretic, Urinary infection)	Plaza Central de Corabastos; Plaza de El Carmen; Plaza de La Perseverancia; Plaza de Las Ferias; Plaza de Mercado de Armenia (Quindío); Plaza de Mercado Trinidad-Galán; Plaza de Paloquemao; Plaza del 12 de Octubre; Plaza del 20 de Julio; Plaza del 7 de Agosto; Plaza del Quirigua; Plaza del Restrepo
Algae			
*Caulerpa* sp.	Caulerpa	Animal food (Animal food)*; Human food (Water purifier)*	Plaza de Mercado de Girardot (Cundinamarca)
*Gracilaria* sp.	Gelosa	Digestive system (Constipation); Human food (Food)*	Plaza Central de Corabastos
Amaranthaceae			
*Amaranthus spinosus* L.	Bledo	Non-specific symptoms and general illnesses (Stomach ache); Reproductive system and sexual health (Menstrual colic); Skin and subcutaneous tissue (Healing wounds, Sores)	Plaza del 7 de Agosto
*Beta vulgaris* L.	Acelga	Digestive system (Constipation, Liver problems); Muscular-skelettal system (Arthritis); Urinary system (Urinary infection)	Plaza de Fontibón
*Chenopodium quinoa* Willd.	Quinua / Quinua roja	Infections and infestations (Tuberculosis); Muscular-skelettal system (Fractures, Luxation, Sprain); Non-specific symptoms and general illnesses (Fever, Stomach ache)Reproductive system and sexual health (Dysmenorrhea)Urinary system (Diuretic)	Plaza Central de Corabastos; Plaza de La Concordia
*Dysphania ambrosioides* (L.) Mosyakin & Clemants	Flor del Paico / Paico / Payco	Blood and circulatory system (Tachycardia); Digestive system (Cholera, Colic, Diarrhea, Digestive problems, Flatulence, Indigestion, Stomach problems); Infections and infestations (Malaria, Vermifuge); Muscular-skelettal system (Arthritis); Non-specific symptoms and general illnesses (Inflammation, Spasms); Reproductive system and sexual health (Emenagogue); Respitarory system (Asthma, Cough, Flu)	Plaza Boyacá; Plaza Central de Corabastos; Plaza de Kennedy; Plaza de Las Ferias; Plaza de Mercado de Armenia (Quindío); Plaza de Mercado Trinidad-Galán; Plaza del Quirigua; Plaza del Restrepo; Plaza Samper Mendoza
*Gomphrena globosa* L.	Siempreviva	Digestive system (Dysentery); Non-specific symptoms and general illnesses (Spasms); Reproductive system and sexual health (Dysmenorrhea)Skin and subcutaneous tissue (Burns, Calluses, Skin diseases, Skin spots, Warts)	Plaza de Paloquemao
*Gomphrena serrata* L.	Abrojo	Non-specific symptoms and general illnesses (Fever); Respitarory system (Bronchitis, Cough, Lung diseases, Pneumonia)	Plaza de Fontibón
*Iresine diffusa* Humb. & Bonpl. ex Willd.	Penicilina	Blood and circulatory system (Anemia)	Plaza del 20 de Julio
Amaryllidaceae			
*Allium sativum* L.	Ajo / Ajo macho / Flor de ajo	Blood and circulatory system (Blood cleansing, Circulatory stimulant, High blood pressure); Cultural illnesses (Good luck, Witchcraft); Digestive system (Gastritis, Intestinal infections, Liver problems); Endocrine system (Diabetes); Infections and infestations (Infections, Vermifuge); Metabolism and nutrition (High cholesterol); Muscular-skelettal system (Arthritis, Rheumatism)Non-specific symptoms and general illnesses (Analgesic, Cancer, Hemorrhage)Others (Tonic); Respitarory system (Bronchitis, Cough)	Plaza de El Carmen; Plaza de Paloquemao; Plaza de San Carlos; Plaza del 12 de Octubre; Plaza del 20 de Julio; Plaza del 7 de Agosto; Plaza del Lucero; Plaza del Restrepo; Plaza Samper Mendoza
*Hippeastrum puniceum* (Lam.) Kuntze	Duende	Cultural illnesses (Witchcraft)	Plaza de Las Cruces; Plaza de Mercado Trinidad-Galán
Anacardiaceae			
*Anacardium occidentale* L.	Cajú / Marañón	Blood and circulatory system (Anemia); Cultural illnesses (Strengthen); Dental health (Mouth infections); Digestive system (Constipation); Endocrine system (Diabetes); Metabolism and nutrition (Scurvy)Nervous system and mental health (Stimulant)Non-specific symptoms and general illnesses (General malaise); Others (Memory, Tonic); Reproductive system and sexual health (Infertility, Sexual potency); Respitarory system (Expectorant, Flu); Skin and subcutaneous tissue (Acne, Calluses, Healing, Skin diseases, Warts); Urinary system (Prostate); Human food (Food)*	Plaza de Kennedy; Plaza de La Concordia; Plaza de Mercado del Municipio de Pacho (Cundinamarca); Plaza de Paloquemao; Plaza del 20 de Julio; Plaza del 7 de Agosto; Plaza del Restrepo
*Mangifera indica* L.	Mango	Dental health (Afts, Mouth infections, Strengthen the gums, Toothache); Infections and infestations (Malaria); Non-specific symptoms and general illnesses (Fever, Inflammation)	Plaza de Paloquemao; Plaza del 20 de Julio
*Schinus molle* L.	Pimiento	Digestive system (Diarrhea); Muscular-skelettal system (Arthritis, Rheumatism); Nervous system and mental health (Stimulant); Sensory system (Conjunctivitis); Urinary system (Diuretic); Human food (Food)*	Plaza de Mercado del Municipio de Pacho (Cundinamarca)
*Spondias purpurea* L.	Ciruela del País	Digestive system (Diarrhea); Skin and subcutaneous tissue (Astringent)	Plaza del Restrepo
Annonaceae			
*Annona cherimola* Mill.	Chirimoyo	Digestive system (Constipation); Non-specific symptoms and general illnesses (Cancer)	Plaza de Paloquemao
*Annona muricata* L.	Guanábana / Guanábano	Cultural illnesses ("Fríos encajados"); Digestive system (Constipation, Diarrhea, Dysentery, Flatulence, Indigestion, Stomach problems, Vomitive); Infections and infestations (Bot fly); Non-specific symptoms and general illnesses (Spasms, Stomach ache); Respitarory system (Flu); Skin and subcutaneous tissue (Bruises); Urinary system (Urinary infection); Human food (Beberage, Food)*	Plaza Boyacá; Plaza de La Perseverancia; Plaza de Mercado Trinidad-Galán; Plaza del 20 de Julio
*Annona squamosa* L.	Anón	Skin and subcutaneous tissue (Bruises); Toxic (Insecticide)*	Plaza del 7 de Agosto
*Xylopia aromatica* (Lam.) Mart.	Malagusta	Digestive system (Strengthens digestive system); Non-specific symptoms and general illnesses (Lack of appetite, Stomach ache); Reproductive system and sexual health (Emenagogue); Skin and subcutaneous tissue (Astringent)	Plaza de Las Cruces
Apiaceae			
*Anethum graveolens* L.	Eneldo	Digestive system (Flatulence, Indigestion); Nervous system and mental health (Stimulant);Human food (Condiment)*	Plaza Central de Corabastos; Plaza de Mercado de Girardot (Cundinamarca); Plaza de Paloquemao; Plaza del Restrepo
*Angelica archangelica* L.	Angélica	Blood and circulatory system (Blood cleansing); Muscular-skelettal system (Rheumatism); Non-specific symptoms and general illnesses (Cancer, Spasms)	Plaza del 20 de Julio
*Apium graveolens* L.	Apio	Blood and circulatory system (Blood cleansing); Digestive system (Colic, Diarrhea, Flatulence, Gallbladder, Indigestion, Liver problems); Muscular-skelettal system (Rheumatism); Nervous system and mental health (Nerves, Stimulant); Non-specific symptoms and general illnesses (Fever, Inflammation, Stomach ache); Others (Sunstroke); Reproductive system and sexual health (Menstrual colic); Respitarory system (Aphonia); Skin and subcutaneous tissue (Healing); Urinary system (Diuretic)	Plaza de Paloquemao; Plaza del 7 de Agosto; Plaza del Lucero
*Arracacia xanthorrhiza* Bancr.	Arracacha	Sensory system (Conjunctivitis)	Plaza de La Perseverancia
*Eryngium foetidum* L.	Cilantrón / Cilantrón / Piurená	Digestive system (Flatulence, Indigestion, Stomach problems); Infections and infestations (Smallpox, Vermifuge); Respitarory system (Flu);Human food (Condiment)*	Plaza Central de Corabastos; Plaza de Kennedy; Plaza de La Perseverancia; Plaza de Mercado de Armenia (Quindío); Plaza de Mercado de Girardot (Cundinamarca); Plaza de Mercado Trinidad-Galán; Plaza del 20 de Julio; Plaza del Restrepo; Plaza Samper Mendoza
*Foeniculum vulgare* Mill.	Hinojo	Digestive system (Colic, Flatulence, Indigestion, Stomach problems, Vomitive); Infections and infestations (Vermifuge); Metabolism and nutrition (Restorative); Nervous system and mental health (Tranquilizer); Non-specific symptoms and general illnesses (Dizziness, Headache, Hemorrhage, Lack of appetite, Spasms); Pregnancy, childbirth and child-bed (Galactogogue, Infected breasts); Reproductive system and sexual health (Emenagogue); Respitarory system (Cough, Expectorant, Flu); Sensory system (Conjunctivitis); Urinary system (Diuretic, Kidney stones); Human food (Condiment, Food)*	Plaza de Mercado de Girardot (Cundinamarca); Plaza de Mercado Trinidad-Galán; Plaza de Paloquemao; Plaza de San Carlos; Plaza del 20 de Julio; Plaza del Quirigua; Plaza del Restrepo
*Niphogeton ternata* (Willd. ex Schltr.) Mathias & Constance	Apio de monte	Digestive system (Colitis, Diarrhea, Dysentery)Muscular-skelettal system (Rheumatism)	Plaza de Las Cruces
*Petroselinum crispum* (Mill.) Nyman ex A.W. Hill	Perejil	Blood and circulatory system (Heart diseases); Digestive system (Flatulence); Reproductive system and sexual health (Dysmenorrhea, Emenagogue);Human food (Condiment)*	Plaza de Mercado Trinidad-Galán
Pimpinella anisum L.	Anís	Blood and circulatory system (Cardiac stimulant, Circulatory stimulant); Digestive system (Flatulence, Indigestion); Nervous system and mental health (Stimulant); Others (Sunstroke); Pregnancy, childbirth and child-bed (Galactogogue); Respitarory system (Help breathing); Urinary system (Diuretic)	Plaza Central de Corabastos; Plaza del 12 de Octubre; Plaza del 20 de Julio
Apocynaceae			
*Allamanda cathartica* L.	Capitana / Copa de Oro / Capitana / Copa de Oro / Jazmín	Anti-venom (Antidote); Digestive system (Constipation); Muscular-skelettal system (Arthritis, Rheumatism); Non-specific symptoms and general illnesses (Headache); Respitarory system (Flu)	Plaza Central de Corabastos; Plaza del 20 de Julio
*Aspidosperma polyneuron* Müll. Arg.	Quimulá / Cumulá	Non-specific symptoms and general illnesses (Apena); Respitarory system (Asthma, Expectorant)	Plaza de Mercado del Municipio de Pacho (Cundinamarca)
*Aspidosperma quebracho-blanco* Schltdl.	Quebracho Blanco	Respitarory system (Asthma, Expectorant)	Plaza de San Carlos
*Couma macrocarpa* Barb. Rodr.	Jansoco	Pregnancy, childbirth and child-bed (Care umbilical cord)	Plaza de Mercado del Municipio de Pacho (Cundinamarca)
*Rauvolfia tetraphylla* L.	Mirto	Anti-venom (Antidote); Blood and circulatory system (High blood pressure)	Plaza de Las Cruces; Plaza de Mercado del Municipio de Pacho (Cundinamarca); Plaza de Paloquemao; Plaza de Kennedy; Plaza de San Benito
*Thevetia peruviana* (Pers.) K. Schum.	Pepa de cabrito / Caucho	Blood and circulatory system (Heart diseases); Digestive system (Constipation); Non-specific symptoms and general illnesses (Fever)	Plaza Samper Mendoza
*Vinca major* L.	Amor antioqueño	Blood and circulatory system (Heart diseases); Digestive system (Constipation)	Plaza Central de Corabastos; Plaza del Restrepo
Araceae			
*Colocasia esculenta* (L.) Schott	Papachina / Pipachin	Non-specific symptoms and general illnesses (Weakness in children); Human food (Food)*	Plaza del 20 de Julio
*Monstera deliciosa* Liebm.	Balazo	Human food (Food)*; Cultural (Anti theft)*	Plaza de Mercado de Girardot (Cundinamarca); Plaza del 7 de Agosto
*Philodendron dyscarpium* R.E. Schult.	Odoká	Human food (Food)*	Plaza Central de Corabastos
*Xanthosoma sagittifolium* (L.) Schott	Mafafa	Human food (Food)*	Plaza Central de Corabastos
Arecaceae			
*Phoenix dactylifera* L.	Datilera	Digestive system (Constipation); Reproductive system and sexual health (Sexual potency); Respitarory system (Expectorant);Human food (Food)*	Plaza Central de Corabastos
Aristolochiaceae			
*Aristolochia grandiflora* Sw.	Bejuco de Carare	Anti-venom (Antidote); Blood and circulatory system (Circulatory stimulant); Muscular-skelettal system (Muscular paralysis); Non-specific symptoms and general illnesses (Analgesic); Reproductive system and sexual health (Emenagogue, Infertility); Skin and subcutaneous tissue (Stinging)	Plaza de Mercado de Girardot (Cundinamarca)
*Aristolochia maxima* Jacq.	Bejuco de Carare / Carare / Guaco	Anti-venom (Antidote); Blood and circulatory system (Circulatory stimulant); Nervous system and mental health (Tranquilizer); Non-specific symptoms and general illnesses (Analgesic); Reproductive system and sexual health (Emenagogue); Skin and subcutaneous tissue (Stinging)	Plaza de El Carmen; Plaza de Kennedy; Plaza de Mercado Trinidad-Galán; Plaza del 12 de Octubre; Plaza del Lucero; Plaza Samper Mendoza
*Aristolochia ringens* Vahl	Guaco	Anti-venom (Antidote); Nervous system and mental health (Sedative); Non-specific symptoms and general illnesses (Analgesic); Reproductive system and sexual health (Emenagogue); Skin and subcutaneous tissue (Stinging)	Plaza de Kennedy
*Aristolochia triangularis* Cham.	Bejuco Carare	Digestive system (Constipation); Non-specific symptoms and general illnesses (Fever); Pregnancy, childbirth and child-bed (Abortive); Reproductive system and sexual health (Emenagogue)	Plaza de La Perseverancia
Asclepiadaceae			
*Asclepias curassavica* L.	Jalapa	Dental health (Toothache); Digestive system (Vomitive); Infections and infestations (Gonorrhea, Vermifuge); Non-specific symptoms and general illnesses (Hemorrhage); Skin and subcutaneous tissue (Skin diseases)	Plaza de Mercado del Municipio de Pacho (Cundinamarca)
*Funastrum clausum* (Jacq.) Schltr.	Bejuco sapo	Digestive system (Vomitive); Sensory system (Conjunctivitis)	Plaza Central de Corabastos
Asparagaceae			
*Cordyline fruticosa* (L.) A. Chev.	Palma morada / Palma real	Urinary system (DiureticUrinary infection)	Plaza Central de Corabastos; Plaza de Las Cruces; Plaza de Mercado de Armenia (Quindío); Plaza del Lucero; Plaza del Restrepo
*Furcraea cabuya* Trel.	Fique / Penca de Fique / Pulque	Blood and circulatory system (Blood cleansing); Digestive system (Hepatic stimulant, Indigestion, Liver problems, Stomach problems); Respitarory system (Flu); Sensory system (Conjunctivitis); Skin and subcutaneous tissue (Skin spots); Urinary system (Diuretic)	Plaza Central de Corabastos; Plaza del 20 de Julio; Plaza del Restrepo
*Furcraea macrophylla* Baker	Fique	Digestive system (Liver problems); Non-specific symptoms and general illnesses (Dropsy); Urinary system (Diuretic)	Plaza del 7 de Agosto
*Sansevieria trifasciata* Prain	Culebrilla	Non-specific symptoms and general illnesses (Menopause); Skin and subcutaneous tissue (Herpes)	Plaza de Fontibón; Plaza de Mercado Trinidad-Galán
Asteraceae			
*Achillea millefolium* L.	Altamisa / Milenrama	Blood and circulatory system (Blood cleansing); Digestive system (Gallbladder, Gastritis, Indigestion, Strengthens digestive system); Infections and infestations (Bot fly); Non-specific symptoms and general illnesses (Analgesic, Hemorrhage, Inflammation, Lack of appetite, Nosebleed, Spasms); Others (Tonic); Reproductive system and sexual health (Emenagogue, Menstrual colic); Skin and subcutaneous tissue (Acne, Boils, Bruises, Healing, Healing wounds, Skin ulcers, Sores); Urinary system (Hemorrhoids)	Plaza de La Perseverancia; Plaza de Las Ferias
*Achyrocline bogotensis* (Kunth) DC.	Vira Vira	Reproductive system and sexual health (Ovarian diseases); Skin and subcutaneous tissue (Rashes, Skin diseases); Urinary system (Prostate, Urethral infections)	Plaza del 20 de Julio; Plaza del Restrepo
*Achyrocline satureioides* (Lam.) DC.	Vira Vira	Non-specific symptoms and general illnesses (Cancer); Skin and subcutaneous tissue (Rashes, Skin diseases)	Plaza de San Benito
*Ambrosia peruviana* Willd.	Altamisa / Artemisa	Cultural illnesses (Witchcraft); Digestive system (Gallbladder, Liver problems); Infections and infestations (Vermifuge); Muscular-skelettal system (Rheumatism); Non-specific symptoms and general illnesses (Analgesic, Hemorrhage, Spasms, Tumors); Others (Tonic); Reproductive system and sexual health (Emenagogue, Menstrual colic); Veterinary (Udder inflammation)	Plaza de Fontibón; Plaza de Las Ferias; Plaza de Mercado de Girardot (Cundinamarca); Plaza de Mercado Trinidad-Galán; Plaza de Paloquemao; Plaza de San Benito; Plaza del 12 de Octubre; Plaza del 20 de Julio; Plaza del 7 de Agosto; Plaza del Restrepo; Plaza Samper Mendoza; Plaza Santander
*Arnica montana* L.	Arnica	Blood and circulatory system (Circulatory stimulant); Dental health (Strengthen the gums); Digestive system (Gallbladder); Muscular-skelettal system (Arthritis, Cramps, Fractures, Luxation, Sprain); Nervous system and mental health (Stimulant); Non-specific symptoms and general illnesses (Analgesic, Dizziness, Inflammation, Promotes sweating, Tumors); Respitarory system (Pharyngitis, Respiratory tract); Skin and subcutaneous tissue (Acne, Astringent, Bruises, Fungicide, Healing, Healing wounds, Skin diseases, Stretch marks)	Plaza de San Benito; Plaza del 7 de Agosto
*Artemisia absinthium* L.	Ajenjo	Blood and circulatory system (Blood cleansing); Cultural illnesses (Affright); Dental health (Halitosis, Toothache); Digestive system (Diarrhea, Flatulence, Gallbladder, Gallstones, Indigestion, Liver problems, Stomach problems, Vomitive); Infections and infestations (Vermifuge); Nervous system and mental health (Tranquilizer); Non-specific symptoms and general illnesses (Analgesic, Headache, Lack of appetite, Stomach ache); Others (Sunstroke, Tonic); Pregnancy, childbirth and child-bed (Breast care); Respitarory system (Cough, Lung diseases, Throat inflamation); Sensory system (Deafness, Otitis); Skin and subcutaneous tissue (Stinging); Urinary system (Diuretic)	Plaza Boyacá; Plaza del 12 de Octubre; Plaza del 20 de Julio; Plaza del 7 de Agosto; Plaza del Quirigua; Plaza del Restrepo; Plaza Santander
*Artemisia vulgaris* L.	Artemisa / Altamisa	Nervous system and mental health (Epilepsy); Pregnancy, childbirth and child-bed (Childbed); Reproductive system and sexual health (Emenagogue)	Plaza de Paloquemao
*Austroeupatorium inulifolium* (Kunth) R.M. King & H. Rob.	Salvia Amarga	Blood and circulatory system (High blood pressure); Digestive system (Liver problems); Non-specific symptoms and general illnesses (Cancer); Respitarory system (Pharyngitis)	Plaza de San Benito; Plaza del Lucero; Plaza del Restrepo
*Baccharis tricuneata* (L. f.) Pers.	Sanalotodo	Endocrine system (Diabetes); Non-specific symptoms and general illnesses (Analgesic)	Plaza del 20 de Julio
*Bidens pilosa* L.	Amor Seco / Chipaca / Masiquia	Cultural illnesses (Witchcraft); Digestive system (Diarrhea, Gallbladder, Indigestion, Liver problems); Endocrine system (Diabetes); Reproductive system and sexual health (Emenagogue); Urinary system (Diuretic, Urinary infection)	Plaza de La Concordia; Plaza de La Perseverancia; Plaza de Las Ferias; Plaza de Mercado Trinidad-Galán; Plaza del 20 de Julio; Plaza del 7 de Agosto; Plaza del Lucero; Plaza del Quirigua; Plaza del Restrepo; Plaza Samper Mendoza
*Calendula officinalis* L.	Caléndula	Blood and circulatory system (Anemia, Blood cleansing); Digestive system (Colon inflammation, Constipation, Duodenum problems, Gastritis, Gastrointestinal disorders, Indigestion); Non-specific symptoms and general illnesses (Analgesic, Dandruff, Hemorrhage, Inflammation, Promotes sweating, Stomach cancer); Reproductive system and sexual health (Menstrual colic); Sensory system (Otitis); Skin and subcutaneous tissue (Skin and subcutaneous tissue, Bruises, Burns, Calluses, Healing, Pustules, Skin diseases, Skin ulcers, Sores, Warts)	Plaza Boyacá; Plaza de El Carmen; Plaza de Fontibón; Plaza de Kennedy; Plaza de La Perseverancia; Plaza de Mercado de Armenia (Quindío); Plaza de Mercado Trinidad-Galán; Plaza de Paloquemao; Plaza de San Carlos; Plaza del 20 de Julio; Plaza del 7 de Agosto; Plaza del Lucero; Plaza del Restrepo; Plaza Samper Mendoza; Plaza Santander
*Chaptalia nutans* (L.) Pol.	Lechuguita	Non-specific symptoms and general illnesses (Hemorrhage); Others (Sunstroke)	Plaza Samper Mendoza
*Chromolaena odorata* (L.) R.M. King & H. Rob.	Rompesaragüey	Non-specific symptoms and general illnesses (Fever, Inflammation, Tumors); Others (Tonic)	Plaza Samper Mendoza
*Chromolaena scabra* (L. f.) R.M. King & H. Rob.	Salvia Amarga	Blood and circulatory system (High blood pressure); Dental health (Mouth diseases); Digestive system (Liver problems, Stomach problems); Non-specific symptoms and general illnesses (Cancer, Mosquito bites, Tumors); Respitarory system (Pharyngitis)	Plaza Central de Corabastos
*Clibadium sylvestre* (Aubl.) Baill.	Barbasco	Infections and infestations (Bot fly); Muscular-skelettal system (Muscular pain);Toxic (Insecticide)*	Plaza de Mercado Trinidad-Galán; Plaza del Restrepo
*Cynara cardunculus* L.	Alcachofa	Blood and circulatory system (Anemia, Arteriosclerosis, Blood cleansing); Digestive system (Constipation, Gallbladder, Hepatic stimulant, Indigestion); Endocrine system (Diabetes); Metabolism and nutrition (Goiter, Restorative); Muscular-skelettal system (Rheumatism); Non-specific symptoms and general illnesses (Weakness in children); Respitarory system (Asthma); Urinary system (Diuretic, Kidney infection, Prostate)	Plaza de La Perseverancia; Plaza de Paloquemao; Plaza del 20 de Julio; Plaza del 7 de Agosto; Plaza del Lucero; Plaza del Restrepo
*Espeletia* aff. *corymbosa* Bonpl.	Frailejón	Blood and circulatory system (Varicose veins); Cultural illnesses ("Fríos encajados"); Pregnancy, childbirth and child-bed (Childbed)	Plaza de Las Ferias
*Espeletia argentea* Bonpl.	Frailejón	Blood and circulatory system (Varicose veins); Pregnancy, childbirth and child-bed (Post partum care); Respitarory system (Asthma, Lung diseases); Sensory system (Otitis)	Plaza de San Carlos
*Espeletiopsis garciae* (Cuatrec.) Cuatrec.	Frailejón	Blood and circulatory system (Varicose veins); Cultural illnesses ("Fríos encajados", Incense for cleanising); Muscular-skelettal system (Arthritis, Rheumatism); Pregnancy, childbirth and child-bed (Childbed); Reproductive system and sexual health (Dysmenorrhea); Respitarory system (Lung diseases); Sensory system (Otitis); Cultural(Detergent)*	Plaza de Mercado Trinidad-Galán
*Galinsoga parviflora* Cav.	Guasca / Guascas	Blood and circulatory system (High blood pressure); Digestive system (Gastrointestinal disorders, Indigestion, Liver problems); Endocrine system (Diabetes); Metabolism and nutrition (Hyperglycemia, Scurvy); Non-specific symptoms and general illnesses (Inflammation); Skin and subcutaneous tissue (Bruises, Healing, Sores); Urinary system (Kidney infection)	Plaza de La Perseverancia; Plaza del 20 de Julio; Plaza del Restrepo
*Gamochaeta americana* (Mill.) Wedd.	Vira Vira	Pregnancy, childbirth and child-bed (Post partum care); Respitarory system (Bronchial diseases, Lung diseases); Urinary system (Urinary infection)	Plaza del 20 de Julio
*Gamochaeta purpurea* (L.) Cabrera	Vira Vira	Non-specific symptoms and general illnesses (Fever, Promotes sweating); Respitarory system (Bronchitis, Expectorant); Skin and subcutaneous tissue (Skin diseases)	Plaza de La Concordia
*Gnaphalium elegans* Kunth	Vira Vira	Non-specific symptoms and general illnesses (Cancer, Hemorrhage, Inflammation); Sinusitis (Prostate, Urinary infection)	Plaza Central de Corabastos; Plaza de Kennedy; Plaza de Mercado Trinidad-Galán; Plaza de Paloquemao; Plaza de San Carlos; Plaza del Restrepo; Plaza Santander
*Gomphrena serrata* L.	Abrojo	Blood and circulatory system (Blood cleansing); Cultural illnesses (Affright); Digestive system (Diarrhea, Digestive problems, Dysentery, Gastritis, Intestinal infections); Non-specific symptoms and general illnesses (Fever, Stomach ache); Others (Tonic); Reproductive system and sexual health (Vaginal discharge); Skin and subcutaneous tissue (Rashes); Urinary system (Urinary infection)	Plaza de San Carlos
*Koanophyllon solidaginoides* (Kunth) R.M. King & H. Rob.	Abrecaminos	Cultural illnesses (Good luck); Digestive system (Flatulence); Respitarory system (Flu, Respiratory tract); Skin and subcutaneous tissue (Rashes, Skin diseases)	Plaza de La Perseverancia; Plaza de Las Cruces; Plaza de Mercado de Girardot (Cundinamarca); Plaza de Mercado Trinidad-Galán; Plaza del Restrepo; Plaza Samper Mendoza
*Matricaria chamomilla* L.	Manzanilla / Matricaria	Human food (Beberage)*; Blood and circulatory system (Cardiac stimulant); Digestive system (Colic, Diarrhea, Flatulence, Indigestion, Stomach problems); Infections and infestations (Infections); Muscular-skelettal system (Arthritis); Nervous system and mental health (Nerves, Stress, Tranquilizer); Non-specific symptoms and general illnesses (Tranquilizer, Non-specific symptoms and general illnesses, Alopecia, Analgesic, Fever, Headache, Inflammation, Promotes sweating, Spasms, Stomach ache); Others (Tonic); Pregnancy, childbirth and child-bed (Breast care); Reproductive system and sexual health (Emenagogue, Menstrual colic); Sensory system (Conjunctivitis); Skin and subcutaneous tissue (Healing, Skin allergies)	Plaza de La Perseverancia; Plaza de Las Cruces; Plaza de Mercado de Armenia (Quindío); Plaza de Mercado Trinidad-Galán; Plaza de Paloquemao; Plaza del 12 de Octubre; Plaza del 20 de Julio; Plaza del 7 de Agosto; Plaza del Quirigua; Plaza del Restrepo; Plaza Samper Mendoza; Plaza de Kennedy
*Mikania guaco* Bonpl.	Guaco	Anti-venom (Antidote); Metabolism and nutrition (Restorative)	Plaza de Paloquemao
*Pentacalia corymbosa* (Benth.) Cuatrec.	Guasquín	Respitarory system (Pharyngitis, Throat inflamation)	Plaza Samper Mendoza
*Senecio formosus* Kunth	Arnica	Muscular-skelettal system (Fractures); Nervous system and mental health (Stimulant); Non-specific symptoms and general illnesses (Inflammation); Skin and subcutaneous tissue (Bruises)	Plaza de Mercado Trinidad-Galán; Plaza de Paloquemao; Plaza de San Carlos
*Silybum marianum* (L.) Gaertn.	Cardo Mariano	Digestive system (Liver problems)	Plaza del Quirigua
*Smallanthus sonchifolius* (Poepp.) H. Rob.	Yacón	Digestive system (Constipation, Gastrointestinal disorders, Indigestion, Intestinal infections, Regenerate intestinal flora); Endocrine system (Diabetes); Metabolism and nutrition (High cholesterol, Hyperglycemia, Lower cholesterol, Obesity, Strengthen inumosystem); Muscular-skelettal system (Osteoporosis); Nervous system and mental health (Nerves); Non-specific symptoms and general illnesses (Colon cancer); Others (Tonic); Human food (Food)*	Plaza de La Concordia; Plaza de Mercado Trinidad-Galán; Plaza del 7 de Agosto; Plaza del Lucero; Plaza del Restrepo
*Spilanthes oppositifolia* (Lam.) D'Arcy	Botón de oro / Botoncillo / Guaca / Chisaca	Cultural illnesses (Good luck); Dental health (Mouth diseases, Mouth infections, Toothache); Digestive system (Gallbladder, Liver problems); Skin and subcutaneous tissue (Healing, Skin spots)	Plaza de Fontibón; Plaza de La Concordia; Plaza de La Perseverancia; Plaza de Paloquemao; Plaza del Restrepo
*Stevia rebaudiana* (Bertoni) Bertoni	Estevia	Blood and circulatory system (Cardiac stimulant, Circulatory stimulant, High blood pressure); Dental health (Cavities); Digestive system (Gastritis, Indigestion); Endocrine system (Diabetes); Metabolism and nutrition (Appetite suppressant, Gout, High cholesterol, Hyperglycemia, Obesity); Nervous system and mental health (Anxiety); Non-specific symptoms and general illnesses (Analgesic); Respitarory system (Flu); Skin and subcutaneous tissue (Fungicide, Healing, Skin care); Urinary system (Diuretic)	Plaza de La Perseverancia; Plaza de Paloquemao; Plaza del 7 de Agosto
*Taraxacum officinale* F.H. Wigg.	Diente de León	Blood and circulatory system (Blood cleansing); Digestive system (Constipation, Gallbladder, Gastritis, Indigestion, Liver problems, Stomach problems); Metabolism and nutrition (Gout, High cholesterol,Obesity); Muscular-skelettal system (Arthritis); Non-specific symptoms and general illnesses (Improve health, Stomach ache); Skin and subcutaneous tissue (Skin ulcers); Urinary system (Diuretic, Urinary infection); Human food (Food)*	Plaza Central de Corabastos; Plaza de La Perseverancia; Plaza de Mercado Trinidad-Galán; Plaza de Paloquemao; Plaza del 20 de Julio; Plaza del 7 de Agosto; Plaza del Restrepo; Plaza Samper Mendoza
Basellaceae			
*Anredera cordifolia* (Ten.) Steenis	Insulina	Endocrine system (Diabetes); Metabolism and nutrition (Hyperglycemia); Muscular-skelettal system (Fractures); Respitarory system (Cough); Sensory system (Conjunctivitis)	Plaza de Paloquemao
*Ullucus tuberosu*s Caldas	Chugua / Ulluco	Digestive system (Constipation); Pregnancy, childbirth and child-bed (Childbed)	Plaza de Mercado de Girardot (Cundinamarca); Plaza del 7 de Agosto
Berberidaceae			
*Berberis glauca* DC.	Tachuelo	Digestive system (Constipation); Non-specific symptoms and general illnesses (Fever, Hemorrhage, Promotes sweating); Others (Tonic)	Plaza del 20 de Julio
*Berberis goudotii* Triana & Planch. ex Wedd.	Espino	Digestive system (Constipation); Infections and infestations (Malaria); Non-specific symptoms and general illnesses (Fever, Hemorrhage); Skin and subcutaneous tissue (Skin Tonic)	Plaza Central de Corabastos; Plaza del Restrepo
Bignoniaceae			
*Crescentia cujete* L.	Totumo	Blood and circulatory system (Blood cleansing, Varicose veins); Non-specific symptoms and general illnesses (Fever, Headache, Hemorrhage); Others (Sunstroke); Pregnancy, childbirth and child-bed (Breast care); Respitarory system (Pregnancy, childbirth and child-bed, Asthma, Bronchial dilator, Bronchial diseases, Cough, Expectorant, Respiratory tract, Sinusitis); Skin and subcutaneous tissue (Skin ulcers)	Plaza Boyacá; Plaza de Las Cruces; Plaza de Las Ferias; Plaza de San Benito; Plaza de San Carlos; Plaza del 20 de Julio; Plaza del Restrepo; Plaza Samper Mendoza
*Delostoma integrifolium* D. Don	Cajeto / Crecedor	Blood and circulatory system (Blood cleansing); Urinary system (Diuretic)	Plaza Samper Mendoza
*Handroanthus barbatus* (E. Mey.) Mattos	Palo de Arco	Digestive system (Gallbladder)	Plaza de La Concordia; Jacaranda caucana Pittier
*Jacaranda caucana* Pittier	Gualanday	Blood and circulatory system (Blood cleansing); Dental health (Mouth infections); Digestive system (Liver problems); Infections and infestations (Anthelmintic, Syphilis, Venereal diseases); Muscular-skelettal system (Bone pain); Non-specific symptoms and general illnesses (Analgesic); Sensory system (Mouth ulcers); Skin and subcutaneous tissue (Rashes, Skin diseases)	Plaza de Kennedy; Plaza de La Perseverancia; Plaza de Las Cruces; Plaza del 20 de Julio; Plaza del 7 de Agosto
*Jacaranda copaia* (Aubl.) D. Don	Gualanday	Blood and circulatory system (Blood cleansing); Infections and infestations (Syphilis, Venereal diseases); Muscular-skelettal system (Arthritis, Neuralgia, Rheumatism); Non-specific symptoms and general illnesses (Analgesic); Skin and subcutaneous tissue (Burns, Skin allergies, Skin ulcers); Urinary system (Urethral infections)	Plaza Boyacá; Plaza de La Perseverancia; Plaza de Mercado Trinidad-Galán; Plaza del Restrepo
*Jacaranda mimosifolia* D. Don	Gualanday	Infections and infestations (Syphilis)	Plaza de Paloquemao
Bixaceae			
*Bixa orellana* L.	Bija / Achiote	Digestive system (Diarrhea, Indigestion, Liver problems); Non-specific symptoms and general illnesses (Cancer, Mosquito bites); Reproductive system and sexual health (Sexual potency); Respitarory system (Bronchitis, Expectorant, Tonsillitis); Skin and subcutaneous tissue (Burns, Eczema, Healing, Skin diseases); Human food (Condiment)*	Plaza de Las Cruces; Plaza de Paloquemao; Plaza de San Benito; Plaza del Quirigua
Bombacaceae			
*Herrania nitida* (Poepp.) R.E. Schult.	Otonasaré	Human food (Food)*	Plaza de Mercado del Municipio de Pacho (Cundinamarca)
Boraginaceae			
*Borago officinalis* L.	Borraja	Blood and circulatory system (Blood cleansing); Cultural illnesses ("Fríos encajados"); Infections and infestations (Measles); Nervous system and mental health (Tranquilizer); Non-specific symptoms and general illnesses (Fever, Inflammation, Promotes sweating); Reproductive system and sexual health (Dysmenorrhea, Emenagogue); Respitarory system (Bronchitis, Cough, Expectorant, Flu); Skin and subcutaneous tissue (Bruises)	Plaza de Kennedy; Plaza de San Carlos; Plaza del 12 de Octubre; Plaza del 20 de Julio; Plaza del 7 de Agosto; Plaza del Restrepo
*Cordia alliodora* (Ruiz & Pav.) Oken	Laurel	Infections and infestations (Infections); Nervous system and mental health (Stimulant); Non-specific symptoms and general illnesses (Promotes sweating)	Plaza del Restrepo
*Cordia dentata* Poir.	Uvito	Non-specific symptoms and general illnesses (Promotes sweating); Respitarory system (Cough, Expectorant)	Plaza de Mercado de Armenia (Quindío)
*Heliotropium arborescens* L.	Heliotropo	Digestive system (Vomitive); Non-specific symptoms and general illnesses (Cancer); Skin and subcutaneous tissue (Skin ulcers)	Plaza de San Carlos
*Symphytum officinale* L.	Confrey	Blood and circulatory system (Arteriosclerosis, Circulatory stimulant, Varicose veins); Digestive system (Diarrhea, Gastritis); Metabolism and nutrition (Gout, Restorative); Muscular-skelettal system (Bone pain, Rheumatism); Non-specific symptoms and general illnesses (Analgesic); Others (Healthy hair); Respitarory system (Bronchitis, Decongestant, Expectorant, Respiratory tract, Sinusitis); Sensory system (Conjunctivitis); Skin and subcutaneous tissue (Burns); Urinary system (Hemorrhoids)	Plaza de El Carmen; Plaza del 20 de Julio; Plaza del Restrepo
Brassicaceae			
*Brassica nigra* (L.) W.D.J. Koch	Mostaza	Blood and circulatory system (Blood cleansing); Digestive system (Indigestion); Metabolism and nutrition (Restorative); Non-specific symptoms and general illnesses (Lack of appetite); Reproductive system and sexual health (Emenagogue); Respitarory system (Bronchitis, Pneumonia)	Plaza del 12 de Octubre
*Brassica rapa* L.	Nabo	Digestive system (Intestinal inflammation); Infections and infestations (Bot fly); Non-specific symptoms and general illnesses (Inflammation); Respitarory system (Asthma, Cough, Respiratory tract)	Plaza de La Perseverancia
*Draba litamo* L. Uribe	Lítamo real	Cultural illnesses (Longevity); Digestive system (Gastrointestinal disorders); Non-specific symptoms and general illnesses (Cancer)	Plaza Central de Corabastos; Plaza de Paloquemao
*Lepidium bipinnatifidum* Desv.	Mastuerzo / Chisgo	Digestive system (Flatulence, Indigestion); Non-specific symptoms and general illnesses (Fever); Others (Alcoholism); Respitarory system (Asthma, Throat inflamation); Skin and subcutaneous tissue (Healing wounds, Skin diseases); Urinary system (Urinary infection)	Plaza del 20 de Julio; Plaza del 7 de Agosto
*Nasturtium officinale* W.T. Aiton	Berros / Mastuerzo	Blood and circulatory system (Anemia); Digestive system (Indigestion, Liver problems); Urinary system (Diuretic);Human food (Food)*	Plaza Central de Corabastos; Plaza de Las Cruces; Plaza del 7 de Agosto; Plaza del Restrepo
*Raphanus sativus* L.	Rábano	Metabolism and nutrition (Obesity)	Plaza Boyacá
Bromeliaceae			
*Tillandsia usneoides* (L.) L.	Melena / Barba de Viejo	Digestive system (Hernia); Muscular-skelettal system (Rheumatism); Urinary system (Hemorrhoids)	Plaza del 7 de Agosto
Burseraceae			
*Bursera graveolens* (Kunth) Triana & Planch.	Palo Santo / Sasafrás / Tatamaco	Blood and circulatory system (Blood cleansing, Circulatory stimulant); Cultural illnesses (Good luck); Digestive system (Hernia); Muscular-skelettal system (Arthritis, Muscular pain, Sprain); Nervous system and mental health (Stress); Non-specific symptoms and general illnesses (Dizziness, Headache, Promotes sweating); Respitarory system (Asthma, Cough, Flu); Skin and subcutaneous tissue (Remove thorns, Skin allergies); Urinary system (Diuretic)	Plaza de Mercado de Girardot (Cundinamarca); Plaza de Paloquemao; Plaza del 7 de Agosto
*Bursera simaruba* (L.) Sarg.	Indio Desnudo / Caratero	Anti-venom (Antidote); Digestive system (Liver problems); Infections and infestations (Gangrene); Metabolism and nutrition (Obesity, Thyroid); Skin and subcutaneous tissue (Remove thorns)	Plaza Samper Mendoza
Cactaceae			
*Opuntia ficus-indica* (L.) Mill.	Nopal / Tuna	Metabolism and nutrition (High cholesterol, Lower cholesterol, Obesity); Non-specific symptoms and general illnesses (Inflammation); Skin and subcutaneous tissue (Spurs on feet); Urinary system (Diuretic);Human food (Food, Water purifier)*	Plaza de La Perseverancia; Plaza del Restrepo
*Pereskia guamacho* F.A.C. Weber	Cactus	Blood and circulatory system (Cardiac stimulant); Digestive system (Liver decongestion); Urinary system (Diuretic)	Plaza del Restrepo
*Tephrocactus molinensis* (Speg.) Backeb.	Tuna	Urinary system (Diuretic); Human food (Food coloring, Water purifier)*	Plaza de Mercado Trinidad-Galán; Plaza del 20 de Julio
Calophyllaceae			
*Mammea americana* L.	Mamey	Infections and infestations (Bot fly); Non-specific symptoms and general illnesses (Alopecia)	Plaza del 20 de Julio
Cannabaceae			
*Cannabis sativa* L.	Marihuana	Cultural illnesses (Hallucinogen); Non-specific symptoms and general illnesses (Stomach cramp); Reproductive system and sexual health (Infertility)	Plaza Boyacá; Plaza del 20 de Julio
Caprifoliaceae			
*Valeriana officinalis* L.	Valeriana	Cultural illnesses (Hypochondria, Vertigo); Dental health (Mouth infections, Toothache); Digestive system (Indigestion); Nervous system and mental health (Anxiety, Epilepsy, Sedative, Tranquilizer); Non-specific symptoms and general illnesses (Headache, Menopause, Seizures, Spasms); Respitarory system (Bronchial diseases, Cough, Throat inflamation)	Plaza de El Carmen; Plaza de Fontibón; Plaza de Kennedy; Plaza de Las Cruces; Plaza de Mercado Trinidad-Galán; Plaza del 20 de Julio; Plaza del 7 de Agosto; Plaza del Quirigua; Plaza del Restrepo; Plaza Santander
Caricaceae			
*Vasconcellea pubescen*s A. DC.	Papayuela	Respitarory system (Cough, Tonsillitis); Skin and subcutaneous tissue (Acne, Warts)	Plaza de Mercado del Municipio de Pacho (Cundinamarca); Plaza del Quirigua
Celastraceae			
*Maytenus laevis* Reissek	Chuchuguaza / Chuchuhuaza	Blood and circulatory system (Anemia); Digestive system (Diarrhea); Metabolism and nutrition (Restorative); Muscular-skelettal system (Arthritis, Rheumatism); Non-specific symptoms and general illnesses (Inflammation, Spasms, Tumors); Reproductive system and sexual health (Sexual potency); Urinary system (Diuretic)	Plaza Central de Corabastos; Plaza de Mercado Trinidad-Galán; Plaza del 20 de Julio; Plaza del 7 de Agosto; Plaza del Restrepo; Plaza Samper Mendoza
Chrysobalanaceae			
*Chrysobalanus icaco* L.	Icaco	Skin and subcutaneous tissue (Astringent)	Plaza de San Benito
Clusiaceae			
*Clusia alata* Planch. & Triana	Gaque / Vencedora	Cultural illnesses (Good luck); Nervous system and mental health (Tranquilizer); Non-specific symptoms and general illnesses (Headache); Reproductive system and sexual health (Emenagogue); Respitarory system (Flu); Human food (Water purifier)*	Plaza Central de Corabastos; Plaza de Kennedy; Plaza de Mercado Trinidad-Galán; Plaza de San Benito
*Clusia multiflora* Kunth	Chagualo	Digestive system (Constipation); Skin and subcutaneous tissue (Skin diseases)	Plaza Central de Corabastos
Commelinaceae			
*Callisia repens* (Jacq.) L.	Sueldo / Suelda con suelda	Muscular-skelettal system (Arthritis, Rheumatism); Non-specific symptoms and general illnesses (Inflammation)	Plaza Central de Corabastos
*Commelina erecta* L.	Suelda con Suelda	Muscular-skelettal system (Arthritis, Dislocation, Fractures, Luxation, Rheumatism); Non-specific symptoms and general illnesses (Inflammation)	Plaza Boyacá; Plaza de El Carmen; Plaza de La Concordia; Plaza de Paloquemao; Plaza del Restrepo
Convolvulaceae			
*Cuscuta partita* Choisy	Hilo de oro	Digestive system (Constipation, Liver problems)	Plaza de Mercado del Municipio de Pacho (Cundinamarca); Plaza Samper Mendoza
*Ipomoea carnea* Jacq.	Batatilla / Kasisé / Ojo de sapo	Anti-venom (Antidote); Digestive system (Constipation)	Plaza Central de Corabastos; Plaza de Mercado del Municipio de Pacho (Cundinamarca); Plaza del 20 de Julio
*Ipomoea hederifolia* L.	Jalapa	Digestive system (Constipation)	Plaza del Restrepo
Coriariaceae			
*Coriaria ruscifolia* L.	Reventadera / Sansú	Cultural illnesses (Hallucinogen); Digestive system (Diarrhea)	Plaza de Mercado del Municipio de Pacho (Cundinamarca); Plaza del Restrepo
Costaceae			
*Costus villosissimus* Jacq.	Insulina	Endocrine system (Diabetes); Respitarory system (Asthma, Bronchitis, Lung diseases)	Plaza de El Carmen; Plaza de La Perseverancia; Plaza de Las Cruces; Plaza de Mercado de Armenia (Quindío); Plaza de Mercado Trinidad-Galán; Plaza del 20 de Julio
Crassulaceae			
*Kalanchoe daigremontiana* Raym.-Hamet & H. Perrier	Aranto	Non-specific symptoms and general illnesses (Cancer)	Plaza de Paloquemao
*Kalanchoe gastonis-bonnieri* Raym.-Hamet & H. Perrier	Lengua de Suegra	Blood and circulatory system (High blood pressure); Digestive system (Diarrhea); Infections and infestations (Abscesses); Metabolism and nutrition (Strengthen inumosystem); Nervous system and mental health (Relaxant); Non-specific symptoms and general illnesses (Analgesic, Cancer, Hemorrhage, Inflammation, Tumors); Pregnancy, childbirth and child-bed (Reduces uterine contractions); Sensory system (Conjunctivitis); Astringent (Healing, Skin care); Urinary system (Kidney infection)	Plaza de Paloquemao; Plaza del Restrepo
*Kalanchoe pinnata* (Lam.) Pers.	Hoja Santa	Digestive system (Liver problems); Endocrine system (Diabetes); Muscular-skelettal system (Muscle relaxant); Nervous system and mental health (Sedative); Non-specific symptoms and general illnesses (Analgesic, Cancer, Inflammation, Mosquito bites); Respitarory system (Cough); Skin and subcutaneous tissue (Bruises, Burns, Healing wounds, Skin diseases, Skin ulcers)	Plaza del Quirigua
Cucurbitaceae			
*Cucurbita maxima* Duchesne	Ahuyama	Digestive system (Constipation, Liver cleaning); Infections and infestations (Anthelmintic); Muscular-skelettal system (Arthritis)	Plaza de El Carmen
*Momordica charantia* L.	Balsamina / Bocado de Culebra / Subicogé / Balsamina	Cultural illnesses (Good luck, Witchcraft); Digestive system (Constipation, Indigestion, Vomitive); Endocrine system (Diabetes); Infections and infestations (Malaria, Venereal diseases, Vermifuge); Metabolism and nutrition (Hyperglycemia); Non-specific symptoms and general illnesses (Fever, Hemorrhage); Reproductive system and sexual health (Emenagogue); Urinary system (Hemorrhoids)	Plaza de Fontibón; Plaza de La Perseverancia; Plaza de Mercado Trinidad-Galán
*Sechium edule* (Jacq.) Sw.	Cidra / Guatila	Blood and circulatory system (Circulatory stimulant); Digestive system (Stomach problems); Endocrine system (Diabetes); Metabolism and nutrition (Obesity); Non-specific symptoms and general illnesses (Inflammation); Reproductive system and sexual health (Diuretic); Human food (Food)*	Plaza de San Benito; Plaza del 20 de Julio
Cunoniaceae			
*Weinmannia tomentosa* L. f.	Encenillo	Non-specific symptoms and general illnesses (Hemorrhage)	Plaza del 7 de Agosto
Cyclanthaceae			
*Carludovica palmata* Ruiz & Pav.	Iraca	Non-specific symptoms and general illnesses (Hemorrhage)	Plaza Central de Corabastos
Cyperaceae			
*Rhynchospora nervosa* (Vahl) Boeckeler	Tote	Respitarory system (Cough, Flu); Animal food (Bait)*	Plaza de El Carmen; Plaza de Mercado Trinidad-Galán; Plaza de Paloquemao
Dilleniaceae			
*Curatella americana* L.	Chaparro	Blood and circulatory system (High blood pressure); Digestive system (Gallstones); Endocrine system (Diabetes); Metabolism and nutrition (Hyperglycemia); Non-specific symptoms and general illnesses (Inflammation); Skin and subcutaneous tissue (Astringent, Healing wounds, Rashes)	Plaza de Kennedy; Plaza de Paloquemao; Plaza del Restrepo
Dioscoreaceae			
*Dioscorea alata* L.	Ñame	Muscular-skelettal system (Arthritis, Rheumatism);Human food (Food)*	Plaza Central de Corabastos; Plaza de La Perseverancia
Dryopteridaceae			
*Dryopteris wallichiana* (Spreng.) Hyl.	Helecho macho	Infections and infestations (Vermifuge); Metabolism and nutrition (Rickets, Scurvy); Muscular-skelettal system (Rheumatism)	Plaza Boyacá; Plaza Central de Corabastos; Plaza del 20 de Julio; Plaza del Lucero
Equisetaceae			
*Equisetum bogotense* Kunth	Cola de caballo	Blood and circulatory system (Arteriosclerosis, Blood cleansing, Circulatory stimulant, Varicose veins); Dental health (Halitosis); Digestive system (Diarrhea, Dysentery, Indigestion, Liver problems); Metabolism and nutrition (Rickets); Muscular-skelettal system (Muscular pain); Non-specific symptoms and general illnesses (Alopecia, Analgesic, Hemorrhage, Inflammation); Reproductive system and sexual health (Vaginal infections); Respitarory system (Flu, Laryngitis, Lung diseases, Throat inflamation); Skin and subcutaneous tissue (Healing, Sores, Sweating); Urinary system (Cystitis, Diuretic, Kidney infection, Urinary infection)	Plaza Central de Corabastos; Plaza de Fontibón; Plaza de Paloquemao; Plaza del 20 de Julio; Plaza del Lucero; Plaza del Restrepo
*Equisetum giganteum* L.	Cola de caballo	Digestive system (Dysentery); Non-specific symptoms and general illnesses (Alopecia, Hemorrhage); Respitarory system (Lung diseases); Urinary system (Diuretic)	Plaza del Restrepo
Ericaceae			
*Bejaria resinosa* Mutis ex L. f.	Pegapega	Respitarory system (Expectorant)	Plaza del 12 de Octubre
*Cavendishia bracteata* (Ruiz & Pav. ex J. St.-Hil.) Hoerold	Quereme / Uva de Anís / Uvito	Muscular-skelettal system (Rheumatism); Skin and subcutaneous tissue (Astringent)	Plaza Boyacá; Plaza de La Concordia
*Cavendishia quereme* (Kunth) Benth. & Hook. f.	Quereme	Cultural illnesses (Witchcraft); Dental health (Toothache); Muscular-skelettal system (Arthritis, Rheumatism)	Plaza de Mercado del Municipio de Pacho (Cundinamarca)
Erythroxylaceae			
*Erythroxylum coca* Lam.	Kaji / Coca	Nervous system and mental health (Sedative, Stimulant, Tranquilizer); Sensory system (Conjunctivitis)	Plaza de Mercado del Municipio de Pacho (Cundinamarca)
Euphorbiaceae			
*Croton funckianus* Müll. Arg..	Drago	Digestive system (Duodenum problems, Gallbladder); Infections and infestations (Anthelmintic, Malaria); Nervous system and mental health (Stimulant); Non-specific symptoms and general illnesses (Fever)	Plaza Central de Corabastos; Plaza de Paloquemao; Plaza del Restrepo; Plaza Santander
*Croton lechleri* Müll. Arg.	Sangre de Drago	Digestive system (Heartburn); Non-specific symptoms and general illnesses (Hemorrhage); Others (Tonic); Skin and subcutaneous tissue (Fungicide, Healing, Skin ulcers)	Plaza de El Carmen
*Croton malambo* H. Karst.	Malambo	Digestive system (Colitis, Diarrhea, Stomach problems); Muscular-skelettal system (Arthritis, Rheumatism); Reproductive system and sexual health (Dysmenorrhea)	Plaza de Mercado del Municipio de Pacho (Cundinamarca)
*Croton schiedeanus* Schltdl.	Almizclillo	Blood and circulatory system (Arteriosclerosis, Blood cleansing); Digestive system (Liver problems); Urinary system (Urinary infection)	Plaza de La Concordia
*Euphorbia hypericifolia* L.	Pimpinela	Infections and infestations (Gangrene)	Plaza del 20 de Julio
*Euphorbia tithymaloides* L.	Ipecacuana / Itamorreal	Blood and circulatory system (Blood cleansing); Digestive system (Liver problems, Vomitive); Infections and infestations (Syphilis); Reproductive system and sexual health (Infertility); Respitarory system (Expectorant)	Plaza de El Carmen; Plaza de La Perseverancia; Plaza de Mercado del Municipio de Pacho (Cundinamarca); Plaza de Mercado Trinidad-Galán; Plaza del Restrepo
*Hura crepitans* L.	Ceibo	Non-specific symptoms and general illnesses (Promotes sweating); Skin and subcutaneous tissue (Pustules, Sores); Urinary system (Diuretic);Animal food (Bait)*	Plaza Central de Corabastos
Jatropha curcas L.	Piñón / Purga / Purga de Fraile	Digestive system (Constipation, Vomitive); Infections and infestations (Venereal diseases); Sensory system (Conjunctivitis); Skin and subcutaneous tissue (Burns, Rashes, Skin diseases)	Plaza de El Carmen; Plaza de Mercado del Municipio de Pacho (Cundinamarca)
*Jatropha gossypiifolia* L.	Higuerillo	Digestive system (Constipation); Pregnancy, childbirth and child-bed (Breast care); Skin and subcutaneous tissue (Skin Tonic);Food(Food)*; Cosmetic (Shampoo)*	Plaza de Mercado del Municipio de Pacho (Cundinamarca); Plaza de Paloquemao
*Manihot esculenta* Crantz	Yuca	Cultural illnesses (Against of Pedrohernández); Digestive system (Diarrhea); Skin and subcutaneous tissue (Skin diseases)	Plaza de Mercado de Armenia (Quindío)
*Phyllanthus niruri* L.	Viernes Santo	Digestive system (Constipation); Endocrine system (Diabetes); Infections and infestations (Bot fly, Lice); Urinary system (Diuretic);Toxic (Insecticide)*	Plaza de Mercado de Armenia (Quindío)
*Ricinus communis* L.	Higuerilla / Higuerillo	Digestive system (Constipation, Diarrhea, Gastritis); Non-specific symptoms and general illnesses (Promotes sweating); Sensory system (Stye); Skin and subcutaneous tissue (Dry skin, Stinging);Human food (Food)*	Plaza de El Carmen; Plaza Santander
Fagaceae			
*Quercus humboldtii* Bonpl.	Encina / Roble	Cultural illnesses (Bed-wetting); Dental health (Inflammation of the gums, Mouth infections, Toothache); Digestive system (Diarrhea, Flatulence, Gastritis, Liver problems); Infections and infestations (Bot fly); Non-specific symptoms and general illnesses (Headache, Hemorrhage, Nosebleed, Stomach ache); Reproductive system and sexual health (Vaginal discharge); Respitarory system (Pharyngitis, Throat inflamation); Sensory system (Mouth ulcers); Urinary system (Hemorrhoids)	Plaza de Kennedy; Plaza de La Perseverancia
Flacourtiaceae			
*Casearia sylvestris* Sw.	Ratón	Skin and subcutaneous tissue (Pustules, Skin diseases, Skin ulcers, Sores)	Plaza de Mercado del Municipio de Pacho (Cundinamarca)
Gentianaceae			
*Gentiana sedifolia* Kunth	Teresita / Alegría de Páramo	Digestive system (Strengthens digestive system)	Plaza Samper Mendoza
*Gentianella corymbosa* (Kunth) Weaver & Ruedenberg	Genciana	Blood and circulatory system (Anemia); Digestive system (Colic, Flatulence, Indigestion); Infections and infestations (Vermifuge); Muscular-skelettal system (Arthritis, Cramps); Nervous system and mental health (Stimulant); Non-specific symptoms and general illnesses (Lack of appetite, Sickness)	Plaza de Las Ferias; Plaza del 12 de Octubre
Geraniaceae			
*Pelargonium peltatum* (L.) L'Hér.	Geranio	Non-specific symptoms and general illnesses (Hemorrhage); Reproductive system and sexual health (Infertility); Respitarory system (Throat inflamation); Urinary system (Kidney infection)	Plaza de La Concordia
Gesneriaceae			
*Columnea kalbreyeriana* Mast.	Sanguinaria / Sangre de Cristo	Anti-venom (Antidote); Digestive system (Colon); Endocrine system (Diabetes); Muscular-skelettal system (Arthritis, Rheumatism); Non-specific symptoms and general illnesses (Hemorrhage); Reproductive system and sexual health (Emenagogue); Human food (Food coloring)*	Plaza de Fontibón; Plaza de Kennedy; Plaza de Mercado Trinidad-Galán; Plaza del 20 de Julio; Plaza del Restrepo
*Drymonia serrulata* (Jacq.) Mart.	Destrancadera	Cultural illnesses (Good luck); Digestive system (Gastrointestinal washes); Non-specific symptoms and general illnesses (Analgesic, Breast cancer, Inflammation, Lung cancer, Prostate cancer, Uterine cancer); Reproductive system and sexual health (Uterine cysts); Urinary system (Prostate)	Plaza Central de Corabastos; Plaza de Fontibón; Plaza de La Perseverancia; Plaza de Las Cruces; Plaza de Mercado de Girardot (Cundinamarca); Plaza del 20 de Julio; Plaza del Restrepo; Plaza Samper Mendoza
*Kohleria spicata* (Kunth) Oerst.	Tusilla	Non-specific symptoms and general illnesses (Hemorrhage); Urinary system (Diuretic, Urinary infection)	Plaza de Kennedy
Hamamelidaceae			
*Hamamelis virginiana* L.	Hamamelis	Blood and circulatory system (Varicose veins); Muscular-skelettal system (Sprain); Non-specific symptoms and general illnesses (Alopecia); Skin and subcutaneous tissue (Bruises); Urinary system (Hemorrhoids)	Plaza de Kennedy
Heliconiaceae			
*Heliconia hirsuta* L. f.	Bijao	Anti-venom (Antidote); Muscular-skelettal system (Arthritis, Rheumatism)	Plaza de Las Ferias; Plaza de Mercado de Girardot (Cundinamarca)
*Heliconia psittacorum* L. f.	Gallito	Muscular-skelettal system (Arthritis, Muscular paralysis, Rheumatism)	Plaza Central de Corabastos; Plaza del Restrepo
Hepaticae-Marchantiaceae			
*Marchantia polymorpha* L.	Hepática de las Fuentes	Digestive system (Liver problems); Urinary system (Kidney stones)	Plaza Central de Corabastos
Hypericaceae			
*Hypericum androsaemum* L.	Hypérico	Infections and infestations (Vermifuge); Non-specific symptoms and general illnesses (Analgesic); Skin and subcutaneous tissue (Healing); Urinary system (Diuretic)	Plaza de El Carmen
*Hypericum licopodioides* Triana & Planch.	Escobo	Cultural illnesses (Against shyness, Bed-wetting); Muscular-skelettal system (Cramps); Nervous system and mental health (Depression); Non-specific symptoms and general illnesses (Analgesic, Menopause); Reproductive system and sexual health (Emenagogue); Skin and subcutaneous tissue (Acne, Burns, Eczema, Healing, Skin care, Skin diseases, Sores)	Plaza del Restrepo
Iridaceae			
*Sisyrinchium tinctorium* Kunth	Espadilla / Ispaguilla	Blood and circulatory system (Blood cleansing); Digestive system (Constipation, Flatulence, Indigestion); Infections and infestations (Syphilis); Respitarory system (Flu, Pneumonia)	Plaza de San Benito; Plaza del 7 de Agosto
Juglandaceae			
*Juglans neotropica* Diels.	Nogal	Blood and circulatory system (Anemia, Blood cleansing); Cultural illnesses (Good luck); Digestive system (Constipation, Indigestion, Liver problems); Endocrine system (Diabetes); Metabolism and nutrition (High cholesterol, Lower cholesterol); Non-specific symptoms and general illnesses (Alopecia, Analgesic, Anti-agin, Dandruff, Promotes sweating); Others (Healthy hair); Reproductive system and sexual health (Dysmenorrhea, Vaginal discharge, Vaginal infections); Sensory system (Mouth ulcers); Skin and subcutaneous tissue (Astringent, Fungicide, Skin Tonic); Urinary system (Urethral infections, Urinary infection)	Plaza Boyacá; Plaza Central de Corabastos; Plaza de Kennedy; Plaza de Las Cruces; Plaza de Mercado Trinidad-Galán; Plaza del 20 de Julio; Plaza del 7 de Agosto
Lamiaceae			
*Cantinoa colombiana* (Epling) Harley & J.F.B. Pastore	Cardo Santo	Non-specific symptoms and general illnesses (Analgesic, Hemorrhage); Skin and subcutaneous tissue (Healing)	Plaza de Kennedy
*Hyptis capitata* Jacq.	Botón Negro	Non-specific symptoms and general illnesses (Analgesic, Hemorrhage); Skin and subcutaneous tissue (Healing)	Plaza de Las Ferias
*Lavandula angustifolia* Mill.	Alhucema / Lavándula Espliego	Digestive system (Indigestion); Muscular-skelettal system (Rheumatism); Nervous system and mental health (Tranquilizer); Non-specific symptoms and general illnesses (Headache); Reproductive system and sexual health (Emenagogue); Respitarory system (Expectorant)	Plaza de Las Cruces
*Marrubium vulgare* L.	Marrubio / Marrubio blanco	Digestive system (Constipation); Infections and infestations (Gangrene, Tuberculosis); Metabolism and nutrition (Obesity); Non-specific symptoms and general illnesses (Fever, Stomach ache); Reproductive system and sexual health (Emenagogue); Respitarory system (Bronchial diseases, Cough, Expectorant)	Plaza de Fontibón; Plaza de La Perseverancia; Plaza de Paloquemao; Plaza del 7 de Agosto; Plaza Samper Mendoza; Melissa officinalis L.
*Melissa officinalis* L.	Melissa / Toronjil	Blood and circulatory system (Heart diseases); Digestive system (Digestive problems, Flatulence, Indigestion); Nervous system and mental health (Nerves, Sedative, Tranquilizer); Non-specific symptoms and general illnesses (Spasms)	Plaza de La Concordia; Plaza de Las Ferias; Plaza de San Benito; Plaza del 20 de Julio; Plaza del 7 de Agosto; Plaza del Lucero; Plaza Samper Mendoza
*Mentha spicata* L.	Sígueme-Sígueme / Querendona / Yerbabuena	Cultural illnesses (Good luck); Dental health (Toothache); Digestive system (Flatulence, Gallbladder, Indigestion, Liver, Stomach problems); Infections and infestations (Vermifuge); Muscular-skelettal system (Cramps, Muscle relaxant); Nervous system and mental health (Stimulant, Tranquilizer); Non-specific symptoms and general illnesses (Analgesic, Dizziness, Inflammation, Spasms); Skin and subcutaneous tissue (Burns); Human food (Beberage)*	Plaza de Fontibón; Plaza de Mercado de Armenia (Quindío); Plaza de Mercado de Girardot (Cundinamarca); Plaza de Mercado Trinidad-Galán; Plaza del Quirigua; Plaza Samper Mendoza; Plaza Santander
*Mentha* x *piperita* L.	Hierbabuena / Menta / Yerbabuena	Dental health (Toothache); Digestive system (Diarrhea, Digestive problems, Flatulence, Indigestion, Strengthens digestive system); Infections and infestations (Vermifuge); Nervous system and mental health (Nerves, Sedative, Tranquilizer); Non-specific symptoms and general illnesses (Analgesic, Hemorrhage, Stomach ache, Vomit); Reproductive system and sexual health (Menstrual colic); Respitarory system (Asthma)	Plaza de Kennedy; Plaza de La Concordia; Plaza de Paloquemao; Plaza del 20 de Julio; Plaza del Restrepo; Plaza Samper Mendoza
*Minthostachys mollis* (Kunth) Griseb.	Tusilago / Poleo grande	Non-specific symptoms and general illnesses (Headache); Respitarory system (Asthma, Cough, Expectorant, Throat inflamation)	Plaza de La Perseverancia
*Ocimum americanum* L.	Albahaca	Digestive system (Colon); Non-specific symptoms and general illnesses (Stomach ache); Urinary system (Kidney stones)	Plaza del Restrepo
*Ocimum basilicum* L.	Albahaca / Albahaca morada	Blood and circulatory system (Arteriosclerosis, Heart diseases); Cultural illnesses (Bed-wetting, Good luck); Digestive system (Colitis, Flatulence, Hepatic stimulant, Indigestion, Intestinal infections); Infections and infestations (Vermifuge); Metabolism and nutrition (High cholesterol); Muscular-skelettal system (Rheumatism); Nervous system and mental health (Nerves, Stimulant, Tranquilizer); Non-specific symptoms and general illnesses (Analgesic, Headache); Reproductive system and sexual health (Emenagogue); Respitarory system (Bronchial diseases, Bronchitis, Flu, Respiratory tract, Throat inflamation); Sensory system (Conjunctivitis, Ear inflammation, Otitis,Strengthens vision); Urinary system (Diuretic, Urethral infections, Urinary infection)	Plaza de Mercado de Armenia (Quindío); Plaza de Mercado de Girardot (Cundinamarca); Plaza de Mercado Trinidad-Galán; Plaza de Paloquemao; Plaza de San Benito; Plaza de San Carlos; Plaza del 20 de Julio; Plaza del Quirigua; Plaza del Restrepo; Plaza Santander
*Ocimum campechianum* Mill.	Albahaca	Cultural illnesses (Bed-wetting); Digestive system (Flatulence, Gastrointestinal disorders, Intestinal infections); Nervous system and mental health (Tranquilizer); Non-specific symptoms and general illnesses (Analgesic); Respitarory system (Bronchitis, Respiratory tract); Sensory system (Otitis, Strengthens vision); Urinary system (Diuretic)	Plaza de Mercado de Girardot (Cundinamarca); Plaza de Mercado Trinidad-Galán
*Origanum majorana* L.	Mejorana	Blood and circulatory system (Circulatory stimulant); Digestive system (Flatulence, Indigestion, Stomach problems, Strengthens digestive system); Nervous system and mental health (Migraine, Nerves, Stimulant, Tranquilizer); Non-specific symptoms and general illnesses (Spasms, Swollen glands); Reproductive system and sexual health (Emenagogue); Respitarory system (Bronchial diseases, Expectorant); Skin and subcutaneous tissue (Pustules, Sores)	Plaza de Kennedy; Plaza de Mercado Trinidad-Galán; Plaza de Paloquemao; Plaza del 20 de Julio; Plaza del 7 de Agosto
*Origanum vulgare* L.	Orégano	Non-specific symptoms and general illnesses (Inflammation)	Plaza del 7 de Agosto
*Phyla dulcis* (Trevir.) Moldenke	Orozú / Orozul	Blood and circulatory system (High blood pressure); Digestive system (Gastrointestinal disorders, Liver problems); Endocrine system (Diabetes); Non-specific symptoms and general illnesses (Fever, Inflammation); Respitarory system (Cough, Respiratory tract)	Plaza de Kennedy; Plaza de Mercado Trinidad-Galán; Plaza de Paloquemao
*Rosmarinus officinalis* L.	Romero	Blood and circulatory system (Anemia, Heart diseases); Cultural illnesses (Good luck, Hypochondria, Vertigo); Digestive system (Diarrhea, Gastritis); Muscular-skelettal system (Arthritis, Rheumatism); Nervous system and mental health (Nerves, Stimulant, Tranquilizer); Non-specific symptoms and general illnesses (Alopecia, Headache, Spasms,Stomach ache); Others (Memory); Reproductive system and sexual health (Emenagogue); Respitarory system (Asthma, Bronchitis, Cough); Sensory system (Strengthens vision); Skin and subcutaneous tissue (Healing wounds); Human food (Condiment)*	Plaza de Kennedy; Plaza de La Perseverancia; Plaza de Mercado Trinidad-Galán; Plaza del 12 de Octubre
*Salvia* cf. *carnea* Kunth	Salvia	Blood and circulatory system (High blood pressure)	Plaza del 7 de Agosto
*Salvia hispanica* L.	Semilla de Chía	Endocrine system (Diabetes); Metabolism and nutrition (Appetite suppressant, Obesity)	Plaza del 12 de Octubre
*Salvia officinalis* L.	Salvia	Dental health (Mouth diseases); Digestive system (Stomach problems); Nervous system and mental health (Tranquilizer); Non-specific symptoms and general illnesses (Analgesic); Respitarory system (Expectorant, Pharyngitis, Throat inflamation); Skin and subcutaneous tissue (Astringent)	Plaza de Mercado de Girardot (Cundinamarca); Plaza de Mercado Trinidad-Galán
*Salvia palifolia* Kunth	Mastranto	Blood and circulatory system (Arteriosclerosis, High blood pressure); Digestive system (Indigestion); Non-specific symptoms and general illnesses (Headache)	Plaza de Paloquemao
*Salvia scutellarioides* Kunth	Mastranto	Blood and circulatory system (Arteriosclerosis); Dental health (Inflammation of the gums); Digestive system (Flatulence, Gastritis, Indigestion); Endocrine system (Diabetes); Muscular-skelettal system (Lumbago); Non-specific symptoms and general illnesses (Altitude sickness, Analgesic, Menopause, Mosquito bites); Others (Sunstroke); Reproductive system and sexual health (Emenagogue, Vaginal infections); Respitarory system (Pharyngitis); Skin and subcutaneous tissue (Astringent, Burns, Healing, Healing wounds, Skin ulcers, Sores, Stinging, Sweating); Urinary system (Cystitis, Diuretic)	Plaza de Kennedy; Plaza de La Perseverancia
*Satureja brownei* (Sw.) Briq.	Poleo	Cultural illnesses (Bed-wetting); Digestive system (Flatulence, Indigestion, Stomach problems); Respitarory system (Flu); Urinary system (Hemorrhoids);Human food (Condiment)*	Plaza de Kennedy; Plaza de Mercado Trinidad-Galán; Plaza del Lucero; Plaza Samper Mendoza
*Satureja montana* L.	Hierba de San Juan	Digestive system (Diarrhea, Flatulence, Indigestion); Infections and infestations (Vermifuge); Non-specific symptoms and general illnesses (Analgesic, Spasms)	Plaza del Quirigua
*Scutellaria incarnata* Vent.	Alegría	Nervous system and mental health (Depression)	Plaza del Restrepo
*Thymus vulgaris* L.	Tomillo	Dental health (Gum pain, Halitosis, Toothache); Digestive system (Constipation, Flatulence, Gastritis, Indigestion, Strengthens digestive system); Infections and infestations (Vermifuge); Muscular-skelettal system (Rheumatism); Non-specific symptoms and general illnesses (Alopecia, Analgesic, Fatigue, Lack of appetite, Spasms, Stomach cramp); Others (Tonic); Reproductive system and sexual health (Emenagogue, Sexual potency, Vaginal discharge); Respitarory system (Cough, Flu, Sinusitis); Skin and subcutaneous tissue (Cultural illnesses, Fungicide, Skin diseases); Human food (Condiment)*	Plaza de Mercado Trinidad-Galán; Plaza de Paloquemao; Plaza del Quirigua; Plaza Samper Mendoza
Lauraceae			
*Persea americana* Mill.	Aguacate / Hoja de aguacate	Blood and circulatory system (Circulatory stimulant, Heart diseases); Digestive system (Diarrhea, Dysentery, Flatulence, Indigestion); Infections and infestations (Abscesses, Vermifuge); Metabolism and nutrition (Obesity); Muscular-skelettal system (Arthritis, Rheumatism); Nervous system and mental health (Stimulant); Non-specific symptoms and general illnesses (Alopecia, Analgesic, Headache); Others (Healthy hair); Reproductive system and sexual health (Mouth infections, Sexual potency, Sterilize, Vaginal discharge, Vaginal infections); Respitarory system (Bronchial diseases, Flu); Skin and subcutaneous tissue (Astringent, Boils, Fungicide, Skin diseases); Urinary system (Diuretic, Hemorrhoids, Urethral infections); Animal food (Animal food)*; Human food (Food)*; Toxic (Rat poison)*	Plaza de Mercado Trinidad-Galán; Plaza del 20 de Julio; Plaza del 7 de Agosto; Plaza del Lucero; Plaza del Restrepo; Plaza Santander
Leguminosae-Caesalpinioideae			
*Bauhinia tarapotensis* Benth.	Casco de vaca	Digestive system (Regenerate intestinal flora)	Plaza de Mercado Trinidad-Galán; Plaza del Restrepo
*Bauhinia ungulata* L.	Casco de vaca	Endocrine system (Diabetes); Non-specific symptoms and general illnesses (Headache)	Plaza de El Carmen; Plaza Samper Mendoza
*Brownea ariza* Benth.	Palo de cruz	Cultural illnesses (Witchcraft); Digestive system (Constipation); Non-specific symptoms and general illnesses (Hemorrhage); Reproductive system and sexual health (Dysmenorrhea)	Plaza de Mercado Trinidad-Galán
*Brownea rosa-de-monte* P.J. Bergius	Palo de cruz	Digestive system (Constipation); Non-specific symptoms and general illnesses (Hemorrhage); Reproductive system and sexual health (Dysmenorrhea)	Plaza del Restrepo
*Cassia fistula* L.	Cañafístula	Digestive system (Constipation)	Plaza del Restrepo
*Cassia grandis* L. f.	Casia	Digestive system (Digestive problems); Infections and infestations (Syphilis); Non-specific symptoms and general illnesses (Fever); Reproductive system and sexual health (Emenagogue, Menstrual colic); Urinary system (Kidney stones)	Plaza de Kennedy
*Chamaecrista desvauxii* (Collad.) Killip	Cargarrocío	Digestive system (Constipation)	Plaza Santander
*Copaifera pubiflora* Benth.	Copaiba	Digestive system (Constipation); Infections and infestations (Venereal diseases); Non-specific symptoms and general illnesses (Fever); Respitarory system (Bronchial diseases, Bronchitis); Urinary system (Hemorrhoids)	Plaza del 20 de Julio
*Myroxylon balsamum* (L.) Harms	Bálsamo de Tolú	Nervous system and mental health (Stimulant); Non-specific symptoms and general illnesses (Promotes sweating); Respitarory system (Bronchial diseases)	Plaza del 20 de Julio
*Myroxylon peruiferum* L. f.	Bálsamo del Perú	Infections and infestations (Tuberculosis); Nervous system and mental health (Stimulant); Respitarory system (Bronchitis, Laryngitis); Skin and subcutaneous tissue (Healing)	Plaza del 20 de Julio
*Parkinsonia aculeata* L.	Retamo	Non-specific symptoms and general illnesses (Fever); Human food (Mead preservation)*	Plaza Central de Corabastos; Plaza de La Concordia
*Phanera variegata* (L.) Benth.	Casco de Vaca / Pata de Buey	Digestive system (Constipation, Dysentery); Non-specific symptoms and general illnesses (Headache)	Plaza del Restrepo
*Senna alata* (L.) Roxb.	Monteyoco	Digestive system (Constipation); Infections and infestations (Bot fly)	Plaza de San Carlos
*Senna alexandrina* Mill.	Sen	Digestive system (Constipation)	Plaza del 20 de Julio; Plaza del Restrepo
*Senna hirsuta* (L.) H.S. Irwin & Barneby	Bicho de café	Blood and circulatory system (Anemia); Digestive system (Diarrhea, Gallstones, Indigestion); Infections and infestations (Vermifuge); Others (Tonic); Urinary system (Kidney stones)	Plaza del 20 de Julio
*Senna occidentalis* (L.) Link	Bicho / Chilinchile / Brusca	Digestive system (Constipation, Diarrhea, Stomach problems); Infections and infestations (Malaria); Non-specific symptoms and general illnesses (Fever); Respitarory system (Asthma); Urinary system (Prostate); Human food (Food)*	Plaza de El Carmen; Plaza de Kennedy
*Senna pallida* (Vahl) H.S. Irwin & Barneby	Escobito	Blood and circulatory system (Blood cleansing); Infections and infestations (Syphilis)	Plaza Central de Corabastos; Plaza del Restrepo
*Senna reticulata* (Willd.) H.S. Irwin & Barneby	Martín Galvis	Metabolism and nutrition (Obesity)	Plaza Central de Corabastos; Plaza de Mercado Trinidad-Galán; Plaza del 20 de Julio
*Tamarindus indica* L.	Tamarindo	Digestive system (Constipation, Flatulence, Gallbladder, Indigestion, Stomach problems); Infections and infestations (Malaria); Non-specific symptoms and general illnesses (Inflammation); Pregnancy, childbirth and child-bed (Abortive); Respitarory system (Throat inflamation); Skin and subcutaneous tissue (Skin ulcers); Urinary system (Urinary infection); Human food (Food)*	Plaza Central de Corabastos; Plaza del Restrepo
*Tara spinosa* (Feuillée ex Molina) Britton & Rose	Dividivi	Non-specific symptoms and general illnesses (Analgesic); Respitarory system (Sinusitis, Tonsillitis)	Plaza Central de Corabastos; Plaza de San Benito
Leguminosae-Mimosoideae			
*Acacia dealbata* Link	Acacia de la India	Digestive system (Constipation); Metabolism and nutrition (Obesity)	Plaza de Fontibón
*Anadenanthera peregrina* (L.) Speg.	Niopo	Blood and circulatory system (Heart diseases); Non-specific symptoms and general illnesses (Cancer, Hemorrhage, Tumors); Skin and subcutaneous tissue (Herpes)	Plaza Central de Corabastos; Plaza de La Perseverancia
*Entada polystachya* (L.) DC.	Ojo de buey	Blood and circulatory system (Heart diseases); Non-specific symptoms and general illnesses (Hemorrhage)	Plaza Central de Corabastos; Plaza del 7 de Agosto
*Inga densiflora* Benth.	Guamo	Digestive system (Dysentery)	Plaza de Fontibón
*Inga ynga* (Vell.) J.W. Moore	Guaba / Guamo Bejuco / Guamo Santafereño	Digestive system (Diarrhea, Dysentery, Intestinal inflammation); Muscular-skelettal system (Rheumatism); Non-specific symptoms and general illnesses (Dropsy); Skin and subcutaneous tissue (Astringent)	Plaza del Lucero
*Mimosa pudica* L.	Dormidera	Nervous system and mental health (Epilepsy, Tranquilizer)	Plaza de Kennedy
*Piptadenia flava* (Spreng. ex DC.) Benth.	Zarza	Cultural illnesses (Hallucinogen)	Plaza Central de Corabastos; Plaza del Restrepo
*Samanea saman* (Jacq.) Merr.	Samán	Digestive system (Constipation); Nervous system and mental health (Tranquilizer);Animal food (Animal food)*; Human food (Beberage)*	Plaza Central de Corabastos; Plaza de Kennedy
*Vachellia farnesiana* (L.) Wight & Arn.	Acacia de la India	Digestive system (Diarrhea, Indigestion); Infections and infestations (Typhus); Non-specific symptoms and general illnesses (Fever); Respitarory system (Bronchial diseases, Laryngitis); Urinary system (Urinary infection)	Plaza de El Carmen
*Vachellia nilotica* (L.) P.J.H. Hurter & Mabb.	Acacia de la India	Digestive system (Gastrointestinal disorders); Infections and infestations (Scabies); Nervous system and mental health (Tranquilizer); Respitarory system (Laryngitis, Respiratory tract); Skin and subcutaneous tissue (Pustules, Sores)	Plaza Central de Corabastos
Leguminosae-Papilionoideae			
*Abrus precatorius* L.	Chochito de Indio / Bejuco Pronia / Pionía / Chochos	Digestive system (Appendicitis); Infections and infestations (Vermifuge); Respitarory system (Bronchial diseases, Expectorant); Sensory system (Conjunctivitis, Stye);Toxic (Insecticide)*	Plaza Central de Corabastos; Plaza de Paloquemao; Plaza del 12 de Octubre; Plaza del 20 de Julio; Plaza del Restrepo; Plaza Santander
*Cajanus cajan* (L.) Huth	Guandul / Fríjol quinchoncho	Digestive system (Gastritis, Indigestion); Nervous system and mental health (Tranquilizer); Non-specific symptoms and general illnesses (Spasms); Skin and subcutaneous tissue (Skin ulcers); Urinary system (Diuretic)	Plaza del Quirigua
*Desmodium triflorum* (L.) DC.	Pega-Pega	Desmodium triflorum (L.) DC. (Reproductive system and sexual health)	Plaza del Restrepo
*Erythrina edulis* Triana ex Micheli	Chachafruto	Urinary system (Diuretic); Human food (Food)*	Plaza de La Perseverancia; Plaza de Mercado de Girardot (Cundinamarca)
*Erythrina poeppigiana* (Walp.) O.F. Cook	Búcaro / Cámbulo	Blood and circulatory system (Heart diseases); Cultural illnesses (Hallucinogen); Digestive system (Constipation); Nervous system and mental health (Sedative); Others (Sunstroke); Respitarory system (Flu); Human food (Food)*	Plaza de La Concordia; Plaza de Mercado de Girardot (Cundinamarca)
*Erythrina rubrinervia* Kunth	Eritrina / Siriguay	Nervous system and mental health (Tranquilizer)	Plaza Samper Mendoza
*Gliricidia sepium* (Jacq.) Kunth ex Walp.	Mata ratón	Others (Sunstroke); Skin and subcutaneous tissue (Skin diseases);Toxic (Insecticide)*	Plaza de Kennedy; Plaza de La Perseverancia; Plaza Samper Mendoza
*Glycine max* (L.) Merr.	Soya	Animal food (Animal food)*; Human food (food)*	Plaza Central de Corabastos
*Indigofera suffruticosa* Mill.	Curí	Dental health (Salivation control); Infections and infestations (Gonorrhea);Human food (Food coloring)*	Plaza de La Perseverancia; Plaza de Mercado de Girardot (Cundinamarca)
*Medicago sativa* L.	Alfalfa	Blood and circulatory system (Blood cleansing, High blood pressure); Dental health (Cavities, Dental health, Gum pain); Digestive system (Skin and subcutaneous tissue); Metabolism and nutrition (Obesity, Restorative, Rickets); Non-specific symptoms and general illnesses (Hemorrhage); Urinary system (Urinary infection);Human food (Food)*	Plaza de Kennedy; Plaza del 12 de Octubre; Plaza del Restrepo
*Mucuna mutisiana* (Kunth) DC.	Ojo de venado / Pica pica	Digestive system (Cholera, Constipation); Infections and infestations (Vermifuge); Non-specific symptoms and general illnesses (Dropsy); Urinary system (Hemorrhoids)	Plaza Central de Corabastos; Plaza de Kennedy
*Spartium junceum* L.	Retama	Digestive system (Constipation); Endocrine system (Diabetes)	Plaza de San Benito
*Vicia faba* L.	Habas	Skin and subcutaneous tissue (Burns, Healing wounds)	Plaza Santander
Liliaceae			
*Lilium candidum* L.	Azucena	Infections and infestations (Abscesses); Pregnancy, childbirth and child-bed (Cracked breasts); Sensory system (Otitis); Skin and subcutaneous tissue (Bruises, Calluses, Skin spots)	Plaza de Paloquemao; Plaza Samper Mendoza
Linaceae			
*Linum usitatissimum* L.	Linaza	Digestive system (Constipation, Intestinal inflammation); Non-specific symptoms and general illnesses (General malaise); Respitarory system (Cough); Urinary system (Urinary infection)	Plaza de Mercado del Municipio de Pacho (Cundinamarca); Plaza del 12 de Octubre
Loganiaceae			
*Spigelia anthelmia* L.	Lombricera	Infections and infestations (Vermifuge)	Plaza de Mercado del Municipio de Pacho (Cundinamarca)
Loranthaceae			
*Oryctanthus alveolatus* (Kunth) Kuijt	Pajarito	Blood and circulatory system (High blood pressure); Muscular-skelettal system (Fractures, Luxation); Pregnancy, childbirth and child-bed (Childbed); Respitarory system (Expectorant, Lung diseases, Tonsillitis)	Plaza Boyacá; Plaza Central de Corabastos; Plaza del 7 de Agosto
*Psittacanthus calyculatus* (DC.) G. Don	Muérdago / Visco	Blood and circulatory system (High blood pressure); Cultural illnesses (Vertigo); Metabolism and nutrition (Gout, High cholesterol, Lower cholesterol); Nervous system and mental health (Epilepsy, Sedative); Non-specific symptoms and general illnesses (Analgesic, Hemorrhage, Inflammation); Skin and subcutaneous tissue (Healing); Urinary system (Diuretic)	Plaza de La Perseverancia; Plaza del 20 de Julio; Plaza del 7 de Agosto
Lycoperdaceae			
*Calvatia cyathiformis* (Bosc) Morgan	Pedo de Bruja	Skin and subcutaneous tissue (Healing wounds)	Plaza Santander
Lycopodiaceae			
*Lycopodium clavatum* L.	Cacho de Venado	Cultural illnesses (To make children walk (1-3 years)); Skin and subcutaneous tissue (Skin care, Skin Tonic)	Plaza de Las Cruces; Plaza de Mercado de Girardot (Cundinamarca)
Lythraceae			
*Cuphea carthagenensis* (Jacq.) J.F. Macbr.	San Antonio	Blood and circulatory system (Blood cleansing)	Plaza de Mercado del Municipio de Pacho (Cundinamarca); Plaza del Restrepo
*Cuphea dipetala* (L.f.) Koehne	Moradita	Dental health (Mouth diseases); Infections and infestations (Syphilis); Respitarory system (Throat inflamation); Skin and subcutaneous tissue (Astringent)	Plaza de Kennedy; Plaza de Mercado del Municipio de Pacho (Cundinamarca)
Malpighiaceae			
*Byrsonima crassifolia* (L.) Kunth	Peralejo / Barbasco	Anti-venom (Antidote); Digestive system (Diarrhea); Non-specific symptoms and general illnesses (Fever); Skin and subcutaneous tissue (Astringent)	Plaza Samper Mendoza
*Heteropterys riparia* Cuatrec.	Saca-sal / Sígalo	Cultural illnesses (Good luck); Infections and infestations (Gonorrhea); Urinary system (Cystitis)	Plaza de Mercado de Girardot (Cundinamarca); Plaza de Paloquemao; Plaza del 20 de Julio
*Malpighia glabra* L.	Ciruelo de perro	Infections and infestations (Gonorrhea); Skin and subcutaneous tissue (Astringent); Urinary system (Urethral infections)	Plaza Central de Corabastos
Malvaceae			
*Alcea rosea* L.	Malva real	Dental health (To help teething, Toothache); Respitarory system (Bronchial diseases, Lung diseases, Throat inflamation); Sensory system (Conjunctivitis)	Plaza del 20 de Julio
*Gossypium hirsutum* L.	Algodonero / Algodón morado	Digestive system (Dysentery); Non-specific symptoms and general illnesses (Fever); Respitarory system (Lung diseases); Urinary system (Diuretic)	Plaza de Kennedy
*Guazuma ulmifolia* Lam.	Guacimo / Guásimo	Digestive system (Diarrhea); Non-specific symptoms and general illnesses (Inflammation); Others (Healthy hair); Skin and subcutaneous tissue (Burns, Healing wounds, Skin diseases); Urinary system (Diuretic)	Plaza de Kennedy; Plaza de San Carlos
*Hibiscus rosa-sinensis* L.	Té de Ibisco / Cayeno	Digestive system (Constipation, Indigestion); Metabolism and nutrition (Scurvy); Non-specific symptoms and general illnesses (Alopecia, Analgesic, Spasms); Others (Tonic); Reproductive system and sexual health (Emenagogue); Respitarory system (Expectorant); Skin and subcutaneous tissue (Astringent, Skin allergies)	Plaza de Kennedy; Plaza del Restrepo
*Hibiscus sabdariffa* L.	Abutilón	Digestive system (Gallbladder); Metabolism and nutrition (Scurvy); Urinary system (Diuretic);Human food (Beberage)*	Plaza Central de Corabastos; Plaza del Restrepo
*Malachra rudis* Benth.	Malvavisco	Digestive system (Gastritis); Non-specific symptoms and general illnesses (Promotes sweating); Respitarory system (Bronchitis, Cough, Expectorant, Flu)	Plaza del 20 de Julio
*Malva parviflora* L.	Malva	Non-specific symptoms and general illnesses (Fever)	Plaza del 20 de Julio
*Malva sylvestris* L.	Malva / Malva común	Digestive system (Colic, Constipation, Diarrhea, Intestinal inflammation); Nervous system and mental health (Tranquilizer); Respitarory system (Cough, Flu, Lung diseases)	Plaza de Mercado Trinidad-Galán; Plaza del 20 de Julio; Plaza del Restrepo
*Malvaviscus penduliflorus* DC.	Resucitado	Human food (Food)*	Plaza Santander
*Matisia cordata* Bonpl.	Zapote / Chupachupa	Human food (Food)*	Plaza de Kennedy
*Modiola caroliniana* (L.) G. Don	Patechulo	Non-specific symptoms and general illnesses (Analgesic); Skin and subcutaneous tissue (Healing)	Plaza Boyacá; Plaza de Mercado Trinidad-Galán
*Ochroma pyramidale* (Cav. ex Lam.) Urb.	Balso	Non-specific symptoms and general illnesses (Headache); Respitarory system (Flu)	Plaza del 12 de Octubre
*Sida cordifolia* L.	Yerba Amarga	Non-specific symptoms and general illnesses (Analgesic, Inflammation); Skin and subcutaneous tissue (Healing wounds)	Plaza de Fontibón; Plaza de Mercado de Armenia (Quindío)
*Sida glomerata* Cav.	Escoba	Non-specific symptoms and general illnesses (Analgesic); Respitarory system (Expectorant)	Plaza Central de Corabastos; Plaza del 7 de Agosto
*Sparrmannia africana* L. f.	Tilo	Blood and circulatory system (Blood cleansing); Muscular-skelettal system (Feet pain, Rheumatism); Nervous system and mental health (Nerves, Sedative, Tranquilizer); Non-specific symptoms and general illnesses (Fatigue, Headache, Promotes sweating, Spasms, Stomach cramp); Respitarory system (Bronchitis, Decongestant, Flu); Urinary system (Urinary infection)	Plaza de Mercado del Municipio de Pacho (Cundinamarca); Plaza de Paloquemao; Plaza del 20 de Julio; Plaza del Restrepo
*Sterculia apetala* (Jacq.) H. Karst.	Majao	Blood and circulatory system (Cardiac stimulant); Nervous system and mental health (Tonic); Urinary system (Diuretic)	Plaza de Las Cruces
Melastomataceae			
*Arthrostemma ciliatum* Pav. ex D. Don	Caña Agria	Non-specific symptoms and general illnesses (Fever)	Plaza de San Benito
Menispermaceae			
*Abuta grandifolia* (Mart.) Sandw.	Vibuajeiria	Non-specific symptoms and general illnesses (Fever)	Plaza Central de Corabastos
Monimiaceae			
*Peumus boldus* Molina	Boldo	Blood and circulatory system (Blood cleansing); Dental health (Toothache); Digestive system (Constipation, Flatulence, Gallbladder, Indigestion, Liver cleaning, Liver problems); Infections and infestations (Syphilis); Muscular-skelettal system (Rheumatism); Nervous system and mental health (Migraine, Tranquilizer); Non-specific symptoms and general illnesses (Dropsy, Headache, Lack of appetite); Sensory system (Strengthens vision); Skin and subcutaneous tissue (Rashes); Urinary system (Diuretic, Kidney infection)	Plaza de Kennedy; Plaza de La Perseverancia; Plaza del 20 de Julio; Plaza del 7 de Agosto
Moraceae			
*Artocarpus altilis* (Parkinson) Fosberg	Hoja del Pan / Frutapán	Endocrine system (Diabetes); Non-specific symptoms and general illnesses (Analgesic); Skin and subcutaneous tissue (Rashes, Warts);Human food (Food)*	Plaza de Mercado Trinidad-Galán; Plaza del Restrepo
*Brosimum utile* (Kunth) Oken	Avichurí	Respitarory system (Asthma, Bronchitis, Lung diseases); Human food (Food)*	Plaza de Mercado de Girardot (Cundinamarca)
*Cecropia peltata* L.	Guarumo / Yarumo	Respitarory system (Respitarory system (Asthma, Bronchitis, Lung diseases)	Plaza Central de Corabastos
*Ficus carica* L.	Brevo	Blood and circulatory system (High blood pressure); Cultural illnesses (Meanness); Digestive system (Constipation); Pregnancy, childbirth and child-bed (Breast care); Respitarory system (Bronchitis, Cough, Throat inflamation); Skin and subcutaneous tissue (Rashes, Skin care, Warts)	Plaza de La Perseverancia; Plaza de Las Cruces; Plaza del 20 de Julio; Plaza del Quirigua
*Ficus insipida* Willd.	Higuerón	Infections and infestations (Infections and infestations (Anthelmintic, Vermifuge)	Plaza de El Carmen; Plaza del Lucero
*Ficus maxima* Mill.	Higuerón	Infections and infestations (Infections and infestations (Vermifuge)	Plaza Central de Corabastos; Plaza del 20 de Julio
*Maclura tinctoria* (L.) D. Don ex Steud.	Dinde / Mora	Dental health (Dental health (Tooth extraction)	Plaza Central de Corabastos
Moringaceae			
*Moringa oleifera* Lam.	Moringa / Hierba de la Vida	Blood and circulatory system (Anemia,Heart diseases, High blood pressure); Dental health (Inflammation of the gums); Digestive system (Constipation, Diarrhea, Gastritis); Endocrine system (Diabetes); Muscular-skelettal system (Arthritis, Rheumatism); Nervous system and mental health (Epilepsy); Non-specific symptoms and general illnesses (Analgesic, Cancer, Dandruff, Dropsy, General malaise, Headache, Inflammation, Stomach ache); Others (Tonic); Pregnancy, childbirth and child-bed (Galactogogue); Respitarory system (Asthma); Skin and subcutaneous tissue (Healing wounds); Urinary system (Kidney stones); Human food (Food)*	Plaza de Mercado Trinidad-Galán; Plaza del 7 de Agosto; Plaza del Restrepo
Muntingiaceae			
*Muntingia calabura* L.	Chitato	Dental health (Mouth infections); Digestive system (Diarrhea, Dysentery, Vomitive); Infections and infestations (Measles); Non-specific symptoms and general illnesses (Fever, Stomach ache); Respitarory system (Bronchitis, Cough); Skin and subcutaneous tissue (Skin diseases)	Plaza de Las Cruces; Plaza del 7 de Agosto
Musaceae			
*Musa* x *paradisiaca* L.	Colicero / Plátano hartón / Topocho	Digestive system (Diarrhea, Indigestion, Stomach problems); Infections and infestations (Tuberculosis); Metabolism and nutrition (Lactose intolerance, Restorative);Human food (Food)*	Plaza Boyacá; Plaza Central de Corabastos; Plaza de Mercado de Girardot (Cundinamarca)
Myricaceae			
*Morella parvifolia* (Benth.) Parra-Os.	Laurel Cruz	Digestive system (Diarrhea); Nervous system and mental health (Stimulant); Respitarory system (Flu); Skin and subcutaneous tissue (Astringent)	Plaza de Paloquemao
Myristicaceae			
*Otoba novogranatensis* Moldenke	Otoba	Digestive system (Flatulence, Liver problems); Infections and infestations (Leprosy, Scabies, Tuberculosis); Non-specific symptoms and general illnesses (Alopecia); Skin and subcutaneous tissue (Dermatitis, Skin diseases)	Plaza Central de Corabastos; Plaza de Fontibón
Myrtaceae			
*Eucalyptus globulus* Labill.	Ocal / Eucalipto	Blood and circulatory system (Varicose veins); Cultural illnesses (Sahumerio); Endocrine system (Diabetes); Infections and infestations (Malaria); Muscular-skelettal system (Rheumatism); Non-specific symptoms and general illnesses (Fever, Promotes sweating); Respitarory system (Asthma, Bronchitis, Cough, Decongestant, Expectorant, Flu, Laryngitis, Lung diseases, Respiratory tract, Sinusitis, Throat inflamation); Skin and subcutaneous tissue (Sores); Toxic (Insecticide)*	Plaza Central de Corabastos; Plaza de La Perseverancia; Plaza de Mercado del Municipio de Pacho (Cundinamarca); Plaza de San Carlos; Plaza del 20 de Julio; Plaza del Restrepo; Plaza Samper Mendoza
*Eugenia uniflora* L.	Nuez moscada	Digestive system (Stomach problems); Urinary system (Hemorrhoids)	Plaza de Mercado del Municipio de Pacho (Cundinamarca)
*Myrcianthes leucoxyla* (Ortega) McVaugh	Arrayán / Palo de arrayán	Dental health (Toothache); Digestive system (Diarrhea, Digestive problems, Dysentery, Indigestion, Stomach problems); Endocrine system (Diabetes); Metabolism and nutrition (Restorative); Muscular-skelettal system (Arthritis, Rheumatism); Nervous system and mental health (Sedative, Stimulant); Others (Tonic); Sensory system (Deafness); Skin and subcutaneous tissue (Astringent)	Plaza de Kennedy; Plaza de La Perseverancia; Plaza de Mercado Trinidad-Galán; Plaza de Paloquemao; Plaza del Quirigua
*Myrtus communis* L.	Mirto	Non-specific symptoms and general illnesses (Analgesic); Respitarory system (Bronchitis, Lung diseases)	Plaza Santander
*Psidium guajava* L.	Guayaba / Guayaba Biche	Blood and circulatory system (Anemia); Dental health (Toothache); Digestive system (Diarrhea, Dysentery, Indigestion); Endocrine system (Diabetes); Nervous system and mental health (Stimulant); Non-specific symptoms and general illnesses (Alopecia, Dropsy, Spasms); Skin and subcutaneous tissue (Healing wounds, Skin ulcers, Sores)	Plaza Central de Corabastos; Plaza de Fontibón; Plaza de Kennedy; Plaza de Paloquemao; Plaza del 20 de Julio
*Psidium guineense* Sw.	Guayabo cimarrón	Digestive system (Diarrhea); Skin and subcutaneous tissue (Skin ulcers)	Plaza de Paloquemao
*Syzygium jambos* (L.) Alston	Pomarroso	Digestive system (Flatulence, Indigestion)	Plaza de Mercado del Municipio de Pacho (Cundinamarca); Plaza del Lucero
Onagraceae			
*Ludwigia peruviana* (L.) H. Hara	Clavito	Respitarory system (Asthma, Bronchial diseases, Lung diseases, Pneumonia)	Plaza del Restrepo
*Oenothera multicaulis* Ruiz & Pav.	Flor de muerto / Yerba de las 3 flores / Injerta	Muscular-skelettal system (Fractures); Non-specific symptoms and general illnesses (Analgesic, Inflammation); Skin and subcutaneous tissue (Bruises)	Plaza Central de Corabastos; Plaza de San Benito; Plaza del 7 de Agosto
Orchidaceae			
*Vanilla planifolia* Andrews	Vainilla	Blood and circulatory system (Anemia); Reproductive system and sexual health (Sexual potency); Urinary system (Urinary infection);Human food (Condiment, Food)*	Plaza Central de Corabastos
Oxalidaceae			
*Oxalis corniculata* L.	Acedera	Infections and infestations (Vermifuge); Non-specific symptoms and general illnesses (Fever)	Plaza Boyacá
*Oxalis tuberosa* Molina	Ibias u Ocas	Digestive system (Gallbladder, Gastritis); Muscular-skelettal system (Rheumatism); Urinary system (Diuretic);Human food (Food)*	Plaza Samper Mendoza
Papaveraceae			
*Fumaria officinalis* L.	Fumaria	Metabolism and nutrition (Scurvy); Nervous system and mental health (Tranquilizer); Reproductive system and sexual health (Emenagogue); Skin and subcutaneous tissue (Rashes)	Plaza del 12 de Octubre; Plaza del 7 de Agosto
*Papaver somniferum* L.	Flor de amapola / Adormidera	Digestive system (Diarrhea); Nervous system and mental health (Sedative); Non-specific symptoms and general illnesses (Analgesic, Inflammation, Promotes sweating, Spasms, Stomach ache); Others (Sunstroke); Reproductive system and sexual health (Dysmenorrhea); Respitarory system (Expectorant, Respiratory tract); Sensory system (Conjunctivitis)	Plaza de Mercado Trinidad-Galán
Passifloraceae			
*Passiflora ligularis* Juss.	Granadilla	Infections and infestations (Vermifuge); Muscular-skelettal system (Back pain); Nervous system and mental health (Epilepsy); Non-specific symptoms and general illnesses (Fever); Skin and subcutaneous tissue (Bruises)	Plaza de Kennedy
*Turnera ulmifolia* L.	Damiana	Reproductive system and sexual health (Infertility, Sexual potency); Respitarory system (Expectorant); Urinary system (Diuretic)	Plaza Samper Mendoza
Phyllanthaceae			
*Phyllanthus niruri* L.	Viernes Santo	Digestive system (Constipation); Infections and infestations (Bot fly, Lice)	Plaza de Kennedy
Phytolaccaceae			
*Petiveria alliacea* L.	Anamú	Dental health (Cavities, Gum pain, Toothache); Infections and infestations (Vermifuge); Muscular-skelettal system (Arthritis, Rheumatism); Non-specific symptoms and general illnesses (Analgesic, Cancer, Inflammation, Promotes sweating, Seizures, Spasms, Tumors); Pregnancy, childbirth and child-bed (Childbed); Respitarory system (Respiratory tract); Skin and subcutaneous tissue (Pustules, Sores); Urinary system (Diuretic)	Plaza Central de Corabastos; Plaza de Kennedy; Plaza de La Concordia; Plaza de La Perseverancia; Plaza de Mercado de Armenia (Quindío); Plaza de Mercado Trinidad-Galán; Plaza de Paloquemao; Plaza de San Benito; Plaza del 12 de Octubre; Plaza del 20 de Julio; Plaza del 7 de Agosto; Plaza del Quirigua; Plaza del Restrepo; Plaza Samper Mendoza
*Phytolacca bogotensis* Kunth	Cargamanta / Guaba	Blood and circulatory system (Varicose veins); Digestive system (Constipation); Infections and infestations (Scabies, Vermifuge); Muscular-skelettal system (Arthritis, Rheumatism); Non-specific symptoms and general illnesses (Cancer, Inflammation, Tumors); Respitarory system (Decongestant); Urinary system (Diuretic)	Plaza de La Concordia; Plaza de Mercado de Girardot (Cundinamarca); Plaza del 20 de Julio
Pinaceae			
*Pinus patula* Schltdl. & Cham.	Pino / Pino silvestre	Nervous system and mental health (Stimulant); Non-specific symptoms and general illnesses (Analgesic); Respitarory system (Cough, Expectorant, Respiratory tract); Urinary system (Diuretic, Prostate)	Plaza de Kennedy; Plaza del 7 de Agosto; Plaza del Restrepo
Piperaceae			
*Peperomia garcia-barrigana* Trel. & Yunck.	Siempreviva	Cultural illnesses (Good luck); Digestive system (Indigestion); Respitarory system (Flu)	Plaza de La Concordia; Plaza de Mercado Trinidad-Galán
*Piper aduncum* L.	Cordoncillo	Digestive system (Dysentery, Indigestion); Non-specific symptoms and general illnesses (Hemorrhage); Skin and subcutaneous tissue (Astringent, Sores)	Plaza de La Concordia; Plaza de Paloquemao; Plaza del 20 de Julio
*Piper arboreum* Aubl.	Cordoncillo	Digestive system (Dysentery, Indigestion); Non-specific symptoms and general illnesses (Hemorrhage); Skin and subcutaneous tissue (Pustules, Sores)	Plaza de Mercado Trinidad-Galán
*Piper obtusilimbum* C. DC.	Desvanecedora	Blood and circulatory system (Varicose veins); Non-specific symptoms and general illnesses (Cancer, Inflammation, Tumors)	Plaza de Mercado Trinidad-Galán; Plaza del Restrepo
Plantaginaceae			
*Digitalis purpurea* L.	Digital	Blood and circulatory system (Cardiac stimulant); Nervous system and mental health (Epilepsy in children, Tranquilizer); Respitarory system (Asthma); Urinary system (Diuretic)	Plaza de La Perseverancia
*Plantago australis* Lam.	Llantén	Dental health (Mouth inflammations); Digestive system (Diarrhea, Liver cleaning); Respitarory system (Expectorant, Throat inflamation); Skin and subcutaneous tissue (Rashes)	Plaza del 20 de Julio
*Plantago major* L.	Llantén	Dental health (Inflammation of the gums, Mouth inflammations); Digestive system (Diarrhea, Dysentery, Gastritis, Indigestion, Liver problems); Infections and infestations (Bot fly, Erysipelas, Gangrene); Muscular-skelettal system (Arthritis); Non-specific symptoms and general illnesses (Analgesic, Fever,Hemorrhage, Inflammation); Others (Tonic); Respitarory system (Expectorant, Respiratory tract, Throat inflamation); Skin and subcutaneous tissue (Astringent, Healing, Skin ulcers); Urinary system (Hemorrhoids, Kidney infection)	Plaza de Las Ferias; Plaza del 20 de Julio; Plaza del Restrepo; Plaza Samper Mendoza
*Scoparia dulcis* L.	Escobilla	Non-specific symptoms and general illnesses (Fever)	Plaza del 7 de Agosto
Poaceae			
*Axonopus scoparius* (Flüggé) Kuhlm.	Micay / Pasto Micay / Pimckay / Yerba micay	Pregnancy, childbirth and child-bed (Galactogogue); Urinary system (Diuretic)	Plaza Central de Corabastos; Plaza de Kennedy; Plaza de Las Cruces; Plaza del 12 de Octubre
*Cymbopogon citratus* (DC.) Stapf	Limoncillo / Limonaria	Cultural illnesses ("Fríos encajados"); Dental health (Cavities, Tooth cleaning); Digestive system (Flatulence); Infections and infestations (Vermifuge); Nervous system and mental health (Stimulant); Non-specific symptoms and general illnesses (Fever, Promotes sweating); Respitarory system (Flu); Skin and subcutaneous tissue (Skin Tonic); Human food (Beberage)*	Plaza Central de Corabastos; Plaza de La Perseverancia; Plaza de Mercado de Armenia (Quindío); Plaza de Mercado Trinidad-Galán; Plaza de Paloquemao; Plaza del 12 de Octubre; Plaza del 20 de Julio; Plaza del Restrepo; Plaza Santander
*Cynodon dactylon* (L.) Pers.	Grama	Digestive system (Constipation); Nervous system and mental health (Stimulant); Non-specific symptoms and general illnesses (Promotes sweating); Pregnancy, childbirth and child-bed (Anti-abortive); Urinary system (Diuretic, Urinary infection)	Plaza de Las Ferias; Plaza de Mercado Trinidad-Galán; Plaza del Restrepo
*Imperata contracta* (Kunth) Hitchc.	Vende agujas	Urinary system (Cystitis, Urinary infection)	Plaza de Mercado Trinidad-Galán
*Oryza sativa* L.	Arroz	Digestive system (Diarrhea, Intestinal inflammation); Non-specific symptoms and general illnesses (Fever); Skin and subcutaneous tissue (Skin Tonic);Human food (Food)*	Plaza de Mercado de Girardot (Cundinamarca); Plaza Samper Mendoza
*Sporobolus indicus* (L.) R. Br.	Grama	Blood and circulatory system (Blood cleansing); Digestive system (Liver problems); Urinary system (Diuretic, Kidney infection, Urinary infection)	Plaza de Las Cruces
Polygalaceae			
*Monnina salicifolia* Ruiz & Pav.	Tinto	Cultural illnesses ("Fríos encajados", Good luck); Respitarory system (Sinusitis)	Plaza de La Perseverancia; Plaza de Mercado Trinidad-Galán; Plaza del 7 de Agosto
*Polygala paniculata* L.	Sarpoleta / Yerba de la Virgen	Infections and infestations (Malaria)	Plaza del Restrepo
Polygonaceae			
*Rheum officinale* Baill.	Ruibarbo	Digestive system (Constipation, Diarrhea, Flatulence, Gallbladder, Stomach problems); Non-specific symptoms and general illnesses (Lack of appetite, Stomach ache)	Plaza de Kennedy; Plaza del 7 de Agosto; Plaza del Restrepo
*Rheum palmatum* L.	Ruibarbo	Digestive system (Constipation, Digestive problems, Flatulence, Gallbladder, Indigestion,Stomach problems); Nervous system and mental health (Tranquilizer); Non-specific symptoms and general illnesses (Headache; Reproductive system and sexual health (Infertility))	Plaza Central de Corabastos; Plaza de Fontibón; Plaza de San Carlos
*Rumex crispus* L.	Lengua de Vaca / Romaza / Lengüevaca	Digestive system (Constipation, Gallbladder, Liver problems, Stomach problems); Metabolism and nutrition (Scurvy); Muscular-skelettal system (Rheumatism); Reproductive system and sexual health (Infertility); Skin and sub-cutaneous tissue (Adstringent); Human food (Food)*	Plaza Central de Corabastos; Plaza de Fontibón; Plaza del Restrepo
*Rumex obtusifolius* L.	Lengua de Vaca	Digestive system (Constipation, Digestive problems, Gallbladder, Stomach problems); Reproductive system and sexual health (Infertility)	Plaza de Paloquemao; Plaza de San Benito; Plaza del Restrepo
*Ruprechtia ramiflora* (Jacq.) C.A. Mey.	Cargamuchacho / Higuerón	Metabolism and nutrition (Restorative); Non-specific symptoms and general illnesses (Improve health)	Plaza Boyacá
Polypodiaceae			
*Phlebodium aureum* (L.) J. Sm.	Calaguala	Blood and circulatory system (Blood cleansing, High blood pressure); Digestive system (Diarrhea, Indigestion); Nervous system and mental health (Tranquilizer)	Plaza de Fontibón
*Polypodium fimbriatum* Maxon	Helecho	Blood and circulatory system (Blood cleansing)	Plaza de Las Ferias
*Serpocaulon triseriale* (Sw.) A.R. Sm.	Helecho común	Digestive system (Constipation)	Plaza del Lucero
Portulacaceae			
*Portulaca oleracea* L.	Verdolaga	Infections and infestations (Vermifuge); Non-specific symptoms and general illnesses (Fever); Skin and subcutaneous tissue (Burns); Urinary system (Urinary infection);Human food (Food)*	Plaza de La Concordia; Plaza de Las Ferias; Plaza del Lucero; Plaza del Restrepo
Primulaceae			
*Myrsine coriacea* (Sw.) R. Br. ex Roem. & Schult.	Espadero / Cucharo	Skin and subcutaneous tissue (Skin allergies)	Plaza del 7 de Agosto
Pteridaceae			
*Adiantum capillus-veneris* L.	Culantrillo	Reproductive system and sexual health (Emenagogue); Respitarory system (Expectorant); Urinary system (Diuretic)	Plaza del 20 de Julio
Ranunculaceae			
*Ranunculus nubigenus* Kunth ex DC.	Oreja de ratón	Skin and subcutaneous tissue (Warts)	Plaza Central de Corabastos; Plaza del 7 de Agosto
Rhamnaceae			
*Rhamnus purshiana* DC.	Cáscara Sagrada	Blood and circulatory system (Circulatory stimulant); Digestive system (Constipation, Indigestion); Non-specific symptoms and general illnesses (Lack of appetite); Urinary system (Hemorrhoids)	Plaza de Kennedy; Plaza del Lucero
Rosaceae			
*Cydonia oblonga* Mill.	Membrillo	Digestive system (Diarrhea, Strengthens digestive system); Respitarory system (Lung diseases); Sensory system (Conjunctivitis); Skin and subcutaneous tissue (Cracked lips); Urinary system (Hemorrhoids)	Plaza del 7 de Agosto
*Malus pumila* Mill.	Manzana verde	Blood and circulatory system (High blood pressure); Non-specific symptoms and general illnesses (Headache)	Plaza de Paloquemao
Rubiaceae			
*Alibertia patinoi* (Cuatrec.) Delprete & C.H. Perss.	Borojó	Non-specific symptoms and general illnesses (Cancer); Reproductive system and sexual health (Sexual potency); Urinary system (Urinary infection)	Plaza de Kennedy; Plaza del 7 de Agosto
*Carapichea ipecacuanha* (Brot.) L. Andersson	Ipecacuana	Respitarory system (Asthma, Cough, Expectorant, Lung diseases)	Plaza del Restrepo
*Cinchona pubescens* Vahl	Cascarilla / Quina	Digestive system (Diarrhea, Indigestion); Infections and infestations (Malaria); Non-specific symptoms and general illnesses (Analgesic, Fever)	Plaza de Kennedy; Plaza de San Carlos
*Morinda citrifolia* L.	Noni	Blood and circulatory system (High blood pressure); Digestive system (Colon, Duodenum problems, Gastritis); Infections and infestations (Vermifuge); Muscular-skelettal system (Arthritis, Bone pain, Muscular pain, Rheumatism); Non-specific symptoms and general illnesses (Cancer, Fever, General malaise, Stomach ache); Reproductive system and sexual health (Emenagogue, Infertility); Respitarory system (Cough, Decongestant, Respiratory tract); Skin and subcutaneous tissue (Skin diseases); Human food (Food)*	Plaza del 20 de Julio; Plaza del Restrepo
*Uncaria tomentosa* (Willd.) DC.	Uña de Gato	Digestive system (Duodenum problems, Gastritis, Gastrointestinal disorders, Intestinal inflammation, Liver problems, Stomach problems); Infections and infestations (HIV); Metabolism and nutrition (Strengthen inumosystem); Muscular-skelettal system (Arthritis, Rheumatism); Non-specific symptoms and general illnesses (Analgesic, Cancer, Inflammation, Stomach ache); Reproductive system and sexual health (Contraceptives); Skin and subcutaneous tissue (Fungicide, Healing wounds, Herpes); Urinary system (Diuretic, Kidney stones)	Plaza Boyacá; Plaza de Kennedy; Plaza de San Carlos; Plaza del 12 de Octubre; Plaza del 20 de Julio; Plaza del Restrepo; Plaza Santander
Rutaceae			
*Citrus aurantiifolia (*igual a C. maxima) (Christm.) Swingle	Hoja del limón / Limón	Blood and circulatory system (High blood pressure); Digestive system (Cirrhosis, Flatulence, Gallbladder, Heartburn, Indigestion); Muscular-skelettal system (Arthritis, Rheumatism); Nervous system and mental health (Tranquilizer); Non-specific symptoms and general illnesses (Analgesic, General malaise, Headache); Respitarory system (Pleura inflamation, Throat inflamation); Sensory system (Conjunctivitis, Stye); Skin and subcutaneous tissue (Pustules, Sores)	Plaza de Mercado Trinidad-Galán; Plaza del 20 de Julio
*Citrus aurantium* (igual a C. maxima) L.	Naranja ácida / Naranja agria	Digestive system (Gallbladder, Liver problems); Nervous system and mental health (Sedative);Human food (Food)*	Plaza de Kennedy; Plaza de Mercado del Municipio de Pacho (Cundinamarca); Plaza del 20 de Julio
*Citrus limetta* (igual a C. maxima) Risso	Lima	Blood and circulatory system (High blood pressure); Urinary system (Cystitis, Kidney stones, Urinary infection)	Plaza de Mercado del Municipio de Pacho (Cundinamarca); Plaza del 7 de Agosto
*Citrus sinensis* (igual a C. maxima) (L.) Osbeck	Azahar / Naranjo	Blood and circulatory system (Heart diseases); Digestive system (Indigestion); Nervous system and mental health (Sedative); Non-specific symptoms and general illnesses (Headache, Spasms, Stomach ache);Human food (Food)*	Plaza de Mercado Trinidad-Galán; Plaza de Paloquemao; Plaza del 12 de Octubre; Plaza del 20 de Julio; Plaza del 7 de Agosto
*Coleonema album* (Thunb.) Bartl. & H.L. Wendl.	Diosme	Cultural illnesses (Good luck); Toxic (Insecticide)*	Plaza del 7 de Agosto
*Ruta graveolens* L.	Ruda	Cultural illnesses (Good luck); Digestive system (Constipation, Flatulence, Indigestion, Stomach problems); Infections and infestations (Erysipelas, Vermifuge); Muscular-skelettal system (Neuralgia); Nervous system and mental health (Epilepsy); Non-specific symptoms and general illnesses (Promotes sweating, Stomach ache); Reproductive system and sexual health (Emenagogue, Menstrual colic, Strengthen uterus, Uterine diseases); Respitarory system (Cough); Skin and subcutaneous tissue (Rashes)	Plaza Central de Corabastos; Plaza de El Carmen; Plaza de Fontibón; Plaza de Kennedy; Plaza de La Perseverancia; Plaza de Las Cruces; Plaza de Mercado de Girardot (Cundinamarca); Plaza de Mercado Trinidad-Galán; Plaza de Paloquemao; Plaza de San Benito; Plaza de San Carlos; Plaza del 20 de Julio; Plaza del 7 de Agosto; Plaza del Lucero; Plaza del Quirigua; Plaza del Restrepo; Plaza Samper Mendoza; Plaza Santander
*Zanthoxylum rigidum* Humb. & Bonpl. ex Willd.	Uña de Gato	Digestive system (Liver problems)	Plaza de Fontibón; Plaza de Mercado del Municipio de Pacho (Cundinamarca); Plaza del Restrepo
Salicaceae			
*Salix humboldtiana* Willd.	Sauce	Dental health (Cavities); Muscular-skelettal system (Bone pain, Rheumatism); Nervous system and mental health (Sedative); Non-specific symptoms and general illnesses (Alopecia, Analgesic, Fever)	Plaza de Kennedy; Plaza de Las Cruces; Plaza de San Benito; Plaza del 20 de Julio
Sapindaceae			
*Aesculus hippocastanum* L.	Castaño de Indias	Metabolism and nutrition (Obesity); Non-specific symptoms and general illnesses (Inflammation)	Plaza de San Benito
*Paullinia yoco* R.E. Schult. & Killip	Yocó	Metabolism and nutrition (Appetite suppressant); Nervous system and mental health (Stimulant); Non-specific symptoms and general illnesses (Analgesic)	Plaza Samper Mendoza
*Sapindus saponaria* L.	Chumbimba	Anti-venom (Antidote); Skin and subcutaneous tissue (Skin Tonic); Urinary system (Cystitis);Cultural (Detergent)*; Cosmetic (Shampoo)*	Plaza Central de Corabastos
Sapotaceae			
*Chrysophyllum cainito* L.	Caimito	Nervous system and mental health (Stimulant); Non-specific symptoms and general illnesses (Fever, Hemorrhage); Skin and subcutaneous tissue (Astringent, Skin Tonic);Human food (Food)*	Plaza Central de Corabastos; Plaza del 20 de Julio
*Manilkara zapota* (L.) P. Royen	Níspero	Blood and circulatory system (Blood cleansing); Non-specific symptoms and general illnesses (Fever); Urinary system (Diuretic, Kidney infection)	Plaza de La Concordia
*Pouteria sapota* (Jacq.) H.E. Moore & Stearn	Zapote	Respitarory system (Flu); Urinary system (Diuretic)	Plaza de Mercado de Armenia (Quindío)
Scrophulariaceae			
*Alonsoa meridionalis* (L. f.) Kuntze	Cascabelito	Respitarory system (Asthma)	Plaza del Restrepo
*Verbascum virgatum* Stokes	Verbasco / Gordolobo	Digestive system (Diarrhea); Muscular-skelettal system (Cramps); Nervous system and mental health (Tranquilizer); Respitarory system (Asthma, Bronchitis, Cough, Expectorant, Flu, Throat inflamation)	Plaza del 7 de Agosto
Simaroubaceae			
*Quassia amara* L.	Aguasio / Cuasia Amarga / Cuasia	Blood and circulatory system (Circulatory stimulant); Cultural illnesses (Strengthen); Digestive system (Hepatic stimulant, Indigestion); Infections and infestations (Malaria); Non-specific symptoms and general illnesses (Fever, Lack of appetite); Urinary system (Kidney infection); Toxic (Insecticide)*	Plaza de Paloquemao; Plaza Samper Mendoza
*Simarouba amara* Aubl.	Simarruba / Tara	Digestive system (Constipation, Diarrhea); Non-specific symptoms and general illnesses (Fever); Others (Alcoholism); Reproductive system and sexual health (Emenagogue); Sensory system (Ear)	Plaza de Paloquemao; Plaza del Restrepo
Smilacaceae			
*Smilax officinalis* Kunth	Zarzaparrila	Anti-venom (Antidote); Blood and circulatory system (Blood cleansing, Circulatory stimulant); Infections and infestations (Syphilis); Muscular-skelettal system (Rheumatism); Nervous system and mental health (Stimulant); Non-specific symptoms and general illnesses (Apena, Lack of appetite, Menopause, Promotes sweating); Reproductive system and sexual health (Virility); Respitarory system (Asthma, Bronchial diseases, Cough, Lung diseases); Skin and subcutaneous tissue (Boils); Urinary system (Diuretic)	Plaza Boyacá; Plaza Central de Corabastos; Plaza de Kennedy; Plaza de La Perseverancia; Plaza de Mercado Trinidad-Galán; Plaza de Paloquemao; Plaza del 7 de Agosto; Plaza del Quirigua; Plaza del Restrepo
*Smilax siphilitica* Humb. & Bonpl. ex Willd.	Zarcilla	Anti-venom (Antidote); Blood and circulatory system (Blood cleansing); Infections and infestations (Syphilis); Nervous system and mental health (Stimulant); Non-specific symptoms and general illnesses (Menopause); Reproductive system and sexual health (Virility)	Plaza Central de Corabastos
Solanaceae			
*Brugmansia candida* Pers.	Borrachero	Cultural illnesses (To go crazy); Muscular-skelettal system (Arthritis, Rheumatism); Nervous system and mental health (Stimulant); Urinary system (Hemorrhoids);Toxic (Intoxicant)*	Plaza de Mercado Trinidad-Galán; Plaza del 20 de Julio; Plaza del 7 de Agosto; Plaza del Restrepo
*Capsicum annuum* L.	Ají	Blood and circulatory system (Varicose veins); Digestive system (Constipation, Flatulence, Indigestion); Muscular-skelettal system (Arthritis, Lumbago, Neuralgia, Rheumatism, Sprain); Nervous system and mental health (Depression, Stimulant); Non-specific symptoms and general illnesses (Analgesic, Heats up, Promotes sweating); Reproductive system and sexual health (Emenagogue); Respitarory system (Flu, Laryngitis, Tonsillitis); Skin and subcutaneous tissue (Boils, Bruises)	Plaza de La Concordia
*Capsicum rhomboideum* (Dunal) Kuntze	Ají	Infections and infestations (Abscesses); Non-specific symptoms and general illnesses (Lack of appetite)	Plaza de San Carlos
*Cestrum buxifolium* Kunth	Tinto	Infections and infestations (Typhus); Non-specific symptoms and general illnesses (Analgesic); Skin and subcutaneous tissue (Skin diseases)	Plaza de Las Ferias; Plaza del Restrepo
*Cestrum mutisii* Willd. ex Roem. & Schult.	Tinto	Infections and infestations (Typhus)	Plaza Central de Corabastos
*Datura sanguinea* Ruiz & Pav.	Borrachero	Non-specific symptoms and general illnesses (Dropsy)	Plaza del 7 de Agosto
*Datura stramonium* L.	Chamico estramonio	Respitarory system (Asthma, Cough)	Plaza del 20 de Julio
*Mandragora officinarum* Bertol.	Mandrágora	Blood and circulatory system (Blood cleansing); Cultural illnesses (Good luck); Digestive system (Constipation, Vomitive); Muscular-skelettal system (Rheumatism); Non-specific symptoms and general illnesses (Analgesic); Scabies (Tetanus); Reproductive system and sexual health (Infertility, Sexual potency)	Plaza Central de Corabastos; Plaza del 12 de Octubre; Plaza del Restrepo; Plaza Santander
*Nicotiana tabacum* L.	Tabaco	Infections and infestations (Bot fly, Scabies, Tetanus); Non-specific symptoms and general illnesses (Mosquito bites); Skin and subcutaneous tissue (Fungicide);Toxic (Insecticide)*	Plaza de Mercado Trinidad-Galán; Plaza del Restrepo
*Physalis peruviana* L.	Uchuva / Capulí	Blood and circulatory system (Blood cleansing); Infections and infestations (Vermifuge); Respitarory system (Cough, Throat inflamation); Sensory system (Conjunctivitis); Urinary system (Diuretic)	Plaza del 7 de Agosto; Plaza Samper Mendoza
*Solanum melongena* L.	Berenjena	Metabolism and nutrition (High cholesterol, Obesity)	Plaza Samper Mendoza
*Solanum nigrum* L.	Pepamora / Yerbamora	Metabolism and nutrition (Restorative); Non-specific symptoms and general illnesses (Inflammation, Promotes sweating); Others (Tonic); Skin and subcutaneous tissue (Burns, Herpes, Skin ulcers)	Plaza del Restrepo
*Solanum nigrum* L.	Yerbamora	Digestive system (Duodenum problems); Infections and infestations (Gangrene); Non-specific symptoms and general illnesses (Analgesic, Stomach cancer); Skin and subcutaneous tissue (Eczema, Pustules, Rash, Rashes, Skin diseases, Stinging); Urinary system (Hemorrhoids)	Plaza de Paloquemao; Plaza del Restrepo
*Solanum pseudocapsicum* L.	Mirto	Cultural illnesses (Good luck); Non-specific symptoms and general illnesses (Analgesic); Urinary system (Diuretic)	Plaza del 20 de Julio
*Solanum quitoense* Lam.	Lulo de Castilla	Nervous system and mental health (Nerves); Others (Sunstroke); Skin and subcutaneous tissue (Skin diseases, Stinging)	Plaza del Restrepo
Sterculiaceae			
*Guazuma ulmifolia* Lam.	Nacedero, Guásimo / Ñee au	Blood and circulatory system (Heart diseases); Non-specific symptoms and general illnesses (General malaise);Human food (Food)*	Plaza de Mercado del Municipio de Pacho (Cundinamarca)
Symplocaceae			
*Symplocos theiformis* (L. f.) Oken	Fruta de pava / Té de Bogotá	Metabolism and nutrition (Restorative); Human food (Beberage)*	Plaza Central de Corabastos
Tropaeolaceae			
*Tropaeolum tuberosum* Ruiz & Pav.	Cubios	Skin and subcutaneous tissue (Eczema, Herpes); Urinary system (Diuretic, Urinary infection);Human food (Food)*	Plaza Central de Corabastos; Plaza de Paloquemao
Urticaceae			
*Cecropia peltata* L.	Guarumo / Yarumo	Blood and circulatory system (Heart diseases); Digestive system (Gallbladder); Others (Sunstroke); Respitarory system (Asthma, Bronchial diseases, Bronchitis, Lung diseases, Respiratory tract); Urinary system (Diuretic)	Plaza de Las Cruces; Plaza de Paloquemao; Plaza del 20 de Julio; Plaza del Restrepo
*Parietaria debilis* G. Forst.	Palitaria	Urinary system (Diuretic, Urinary infection)	Plaza Central de Corabastos; Plaza de Fontibón; Plaza de Kennedy; Plaza de Mercado Trinidad-Galán; Plaza de Paloquemao; Plaza del 20 de Julio
*Parietaria officinalis* L.	Parietaria	Muscular-skelettal system (Arthritis); Skin and subcutaneous tissue (Healing wounds); Urinary system (Cystitis, Urinary infection)	Plaza del Quirigua
*Urera baccifera* (L.) Gaudich. ex Wedd.	Pringamosa	Infections and infestations (Erysipelas); Non-specific symptoms and general illnesses (Hemorrhage); Respitarory system (Bronchitis, Urinary system (Urethral infections)	Plaza de San Carlos; Plaza del Restrepo
*Urera caracasana* (Jacq.) Gaudich. ex Griseb.	Pringamoza negra	Non-specific symptoms and general illnesses (Hemorrhage)	Plaza de La Concordia
*Urtica dioica* L.	Ortiga / Ortiga mayor	Blood and circulatory system (Blood cleansing, Circulatory stimulant); Digestive system (Stomach problems); Muscular-skelettal system (Arthritis); Non-specific symptoms and general illnesses (Heats up); Reproductive system and sexual health (Emenagogue, Uterus inflammation); Skin and subcutaneous tissue (Skin allergies); Urinary system (Diuretic)	Plaza de Paloquemao; Plaza del 20 de Julio; Plaza del 7 de Agosto
*Urtica* sp.	Poleo silvestre	Skin and subcutaneous tissue (Skin allergies, Skin diseases)	Plaza del Restrepo
*Urtica urens* L.	Ortiga / Ortiga blanca / Ortiga menor	Blood and circulatory system (Varicose veins); Digestive system (Diarrhea, Gastritis); Metabolism and nutrition (Boddy cleansing); Non-specific symptoms and general illnesses (Alopecia, Dandruff, Inflammation, Nosebleed); Reproductive system and sexual health (Uterus inflammation); Respitarory system (Asthma, Expectorant); Skin and subcutaneous tissue (Rashes, Skin allergies, Skin diseases); Urinary system (Diuretic, Kidney infection); Human food (Condiment)*	Plaza Central de Corabastos; Plaza de Kennedy; Plaza de La Concordia; Plaza de Las Ferias; Plaza de Mercado de Girardot (Cundinamarca); Plaza de Mercado Trinidad-Galán; Plaza del 12 de Octubre; Plaza del 20 de Julio; Plaza del 7 de Agosto; Plaza del Restrepo
Verbenaceae			
*Aloysia citriodora* Paláu	Cidrón	Blood and circulatory system (Circulatory stimulant, Heart diseases); Dental health (Toothache); Digestive system (Digestive problems, Flatulence, Gastrointestinal disorders, Indigestion, Stomach problems); Infections and infestations (Rabies); Nervous system and mental health (Depression, Nerves, Sedative, Stress, Tranquilizer); Non-specific symptoms and general illnesses (Seizures, Spasms); Respitarory system (Expectorant)	Plaza Central de Corabastos; Plaza de El Carmen; Plaza de Las Ferias; Plaza de Mercado de Armenia (Quindío); Plaza de Mercado de Girardot (Cundinamarca); Plaza de Mercado Trinidad-Galán; Plaza de Paloquemao; Plaza de San Benito; Plaza del 20 de Julio; Plaza del 7 de Agosto; Plaza del Restrepo; Plaza Santander
*Bouchea prismatica* (L.) Kuntze	Arrocillo	Digestive system (Rectal problems); Infections and infestations (Malaria, Typhus); Non-specific symptoms and general illnesses (Analgesic); Skin and subcutaneous tissue (Skin Tonic)	Plaza Central de Corabastos
*Lantana camara* L.	Sanguinaria / Venturosa / Sanguinaria	Blood and circulatory system (Blood cleansing, High blood pressure, Varicose veins); Digestive system (Indigestion, Liver problems, Stomach problems); Non-specific symptoms and general illnesses (Analgesic, Fever, Promotes sweating, Spasms); Pregnancy, childbirth and child-bed (Breast care); Reproductive system and sexual health (Emenagogue); Respitarory system (Expectorant, Flu); Urinary system (Diuretic)	Plaza Boyacá; Plaza del 20 de Julio; Plaza del Restrepo; Plaza Samper Mendoza
*Lantana canescens* Kunth	Zorrillo	Non-specific symptoms and general illnesses (Fever); Reproductive system and sexual health (Emenagogue, Uterine diseases); Respitarory system (Bronchitis)	Plaza de Mercado de Armenia (Quindío)
*Lantana trifolia* L.	Venturosa	Muscular-skelettal system (Rheumatism); Non-specific symptoms and general illnesses (Promotes sweating); Reproductive system and sexual health (Emenagogue)	Plaza del 12 de Octubre
*Lippia alba* (Mill.) N.E. Br. ex Britton & P. Wilson	Curalotodo / Pronto alivio / Prontoalivio	Digestive system (Colic, Digestive problems, Flatulence, Indigestion, Stomach problems); Endocrine system (Diabetes); Muscular-skelettal system (Arthritis, Bone pain, Rheumatism); Nervous system and mental health (Nerves, Sedative, Tranquilizer); Non-specific symptoms and general illnesses (Analgesic, Fever, Headache, Promotes sweating, Spasms, Stomach ache); Reproductive system and sexual health (Emenagogue, Menstrual colic); Respitarory system (Asthma, Bronchial diseases, Cough, Flu, Sinusitis)	Plaza de El Carmen; Plaza de Fontibón; Plaza de Kennedy; Plaza de La Perseverancia; Plaza de Las Cruces; Plaza de Mercado Trinidad-Galán; Plaza del 12 de Octubre; Plaza del Lucero; Plaza Santander
*Lippia hirsuta* L.f.	Bunquín	Infections and infestations (Tuberculosis); Non-specific symptoms and general illnesses (Spasms); Reproductive system and sexual health (Emenagogue); Respitarory system (Asthma, Bronchitis, Expectorant, Lung diseases)	Plaza Central de Corabastos
*Verbena officinalis* L.	Verbena	Blood and circulatory system (High blood pressure); Digestive system (Diarrhea, Gallbladder, Stomach ache); Infections and infestations (Typhus); Non-specific symptoms and general illnesses (Analgesic, Headache, Tumors); Skin and subcutaneous tissue (Healing wounds)	Plaza de San Carlos; Plaza del Restrepo; Plaza Samper Mendoza
Violaceae			
*Viola odorata* L.	Violeta	Non-specific symptoms and general illnesses (Fever); Respitarory system (Cough)	Plaza de Las Cruces
Vitaceae			
*Cissus verticillata* (L.) Nicolson & C.E. Jarvis	Bejuco de Agua	Cultural illnesses (To make children walk (1-3 years)); Digestive system (Constipation, Digestive problems); Infections and infestations (Venereal diseases); Muscular-skelettal system (Muscular pain, Rheumatism); Non-specific symptoms and general illnesses (Analgesic, Inflammation, Promotes sweating); Pregnancy, childbirth and child-bed (Breast care); Respitarory system (Flu, Respiratory tract); Skin and subcutaneous tissue (Healing); Urinary system (Diuretic, Hemorrhoids)	Plaza de Mercado de Armenia (Quindío); Plaza Samper Mendoza
Winteraceae			
*Drimys granadensis* L. f.	Canelón	Nervous system and mental health (Stimulant); Skin and subcutaneous tissue (Skin Tonic);Human food (Beberage)*	Plaza de Mercado Trinidad-Galán; Plaza de San Carlos
*Drimys winteri* J.R. Forst. & G. Forst.	Canelo	Nervous system and mental health (Stimulant); Others (Tonic)	Plaza del 7 de Agosto
Xanthorrhoeaceae			
*Aloe vera* (L.) Burm. f.	Sábila	Digestive system (Constipation); Infections and infestations (Leprosy); Reproductive system and sexual health (Dysmenorrhea, Emenagogue); Respitarory system (Bronchial diseases, Bronchitis, Cough, Expectorant, Pneumonia); Skin and subcutaneous tissue (Healing, Healing wounds, Skin diseases, Sweating); Toxic (Insecticide)*	Plaza Central de Corabastos; Plaza de El Carmen; Plaza de Kennedy; Plaza de Las Ferias; Plaza de San Benito; Plaza del 7 de Agosto
Zingiberaceae			
*Curcuma longa* L.	Azafrán de raíz / Cúrcuma	Blood and circulatory system (Circulatory stimulant, Thrombosis); Digestive system (Indigestion, Liver cleaning); Endocrine system (Diabetes); Metabolism and nutrition (High cholesterol, Obesity, Strengthen inumosystem); Muscular-skelettal system (Arthritis); Non-specific symptoms and general illnesses (Cancer); Skin and subcutaneous tissue (Healing wounds); Urinary system (Kidney infection); Human food (Condiment)*	Plaza del Restrepo
*Elettaria cardamomum* (L.) Maton	Cardamomo	Digestive system (Digestive problems); Nervous system and mental health (Stimulant); Non-specific symptoms and general illnesses (Lack of appetite); Respitarory system (Expectorant)	Plaza de Paloquemao; Plaza del 12 de Octubre
Zygophyllaceae			
*Bulnesia arborea* (Jacq.) Engl.	Guayacán	Muscular-skelettal system (Rheumatism)	Plaza del Quirigua
*Kallstroemia maxima* (L.) Hook. & Arn.	Abrojo de patio / Verdolaga / Abrojo	Digestive system (Colon, Indigestion); Infections and infestations (Abscesses, Vermifuge); Others (Ceas smoke); Respitarory system (Bronchitis, Cough); Skin and subcutaneous tissue (Astringent, Pustules); Urinary system (Diuretic)	Plaza Boyacá; Plaza de El Carmen; Plaza de Las Cruces; Plaza de Mercado de Girardot (Cundinamarca); Plaza de Mercado Trinidad-Galán; Plaza de Paloquemao; Plaza del 20 de Julio; Plaza del 7 de Agosto; Plaza del Quirigua; Plaza Santander

Most remedies were taken orally as decoctions/infusions, and a much smaller number was applied as cataplasm. Applications involved preferentially leaves and aerial parts of the plant.

Four hundred two species were used for medicinal purposes. The most treated illness categories were Non-specific symptoms and general illnesses (223 species, 55.4%), Digestive system (208, 52%), Skin and subcutaneous tissue (142, 35.3%), Respiratory system (131, 32.6%), Urinary system (119, 29.6%), Blood and circulatory system (107, 26.6%), and Infections and infestations (100, 24.8%). Among the individual illnesses, indigestion (167 species, 41.5%) stood out as the most common illness, followed by diuretic uses (155, 38.5%), emmenagogues (125, 32%), and flatulence (123, 30.5%) and rheumatism, diarrhea, and use as laxative (120, 119, and 166 species, 30, 29.5, and 29%, respectively).

**Fig. 1:**
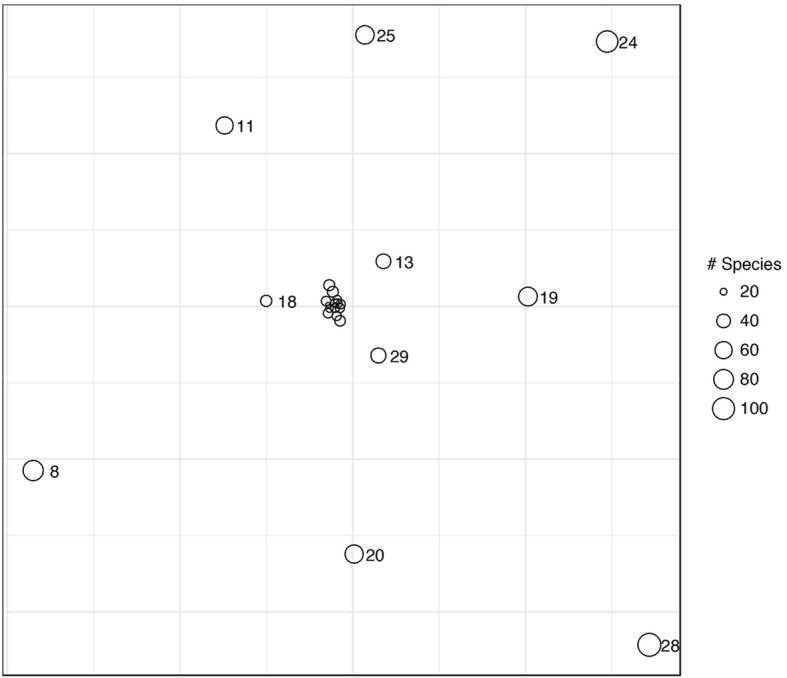
Ordination of Colombian markets in plant-space (plant species and their respective uses). The difference between the plants venders report is not explained by geographical factors (by 9 geographical zones: *p* = 0.37; by 15 localities: *p* = 0.41) or size (by number of species reported: *p* = 0.22)

**Fig. 2:**
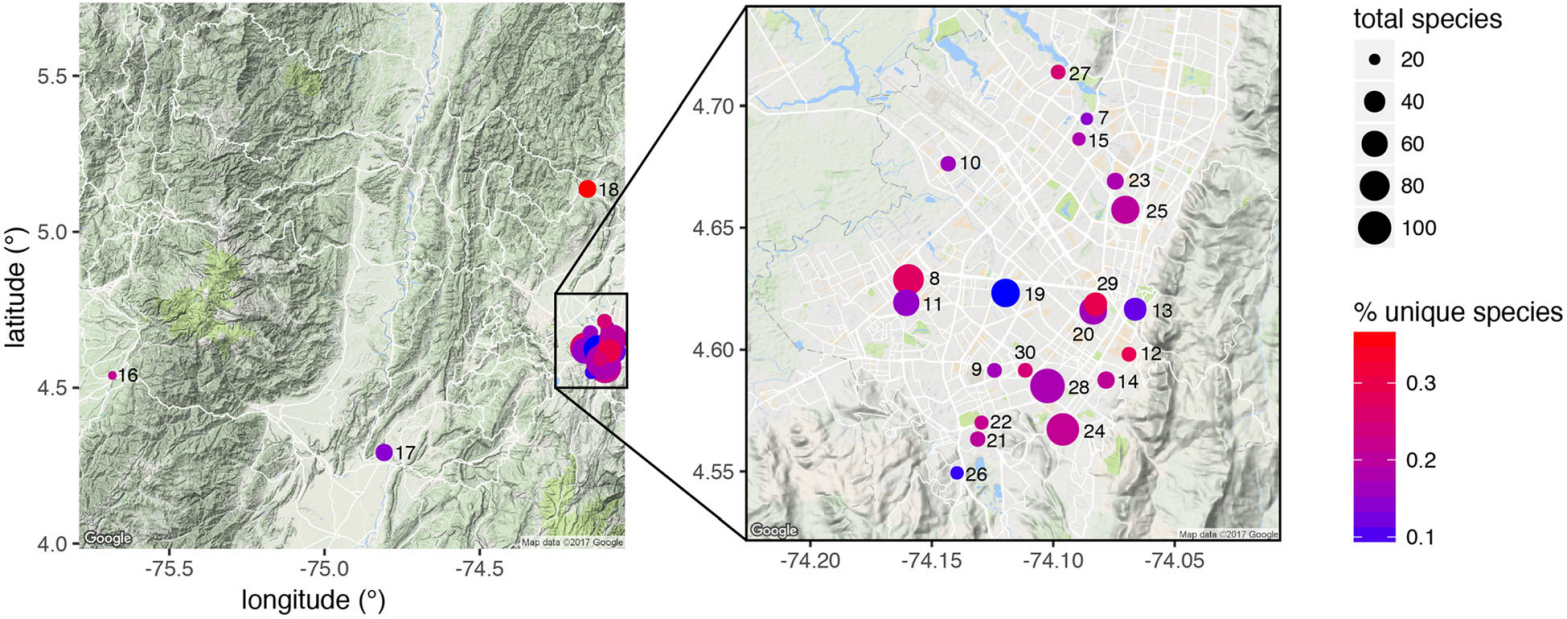
Proportion of unique plants in Colombian markets

**Fig. 3:**
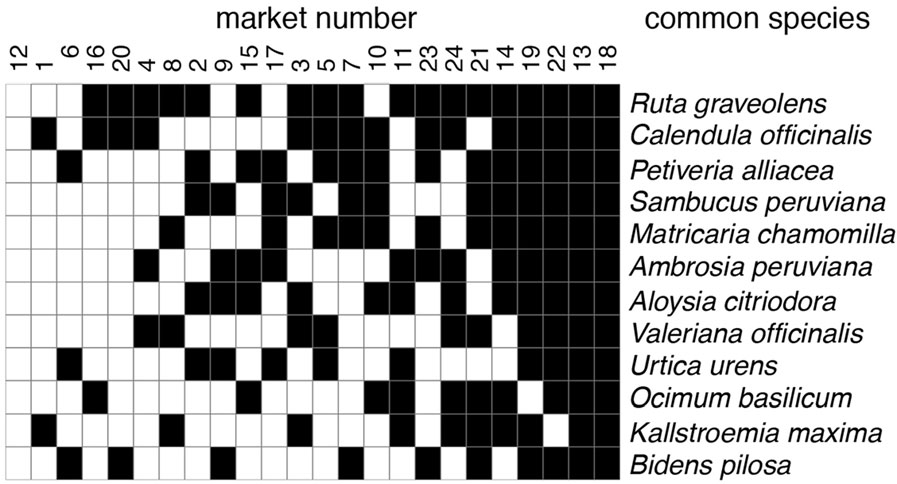
The 11 most widespread species among Colombian markets (black squares)

In the present study, almost without exception, the small markets contained much less species than the main markets. Markets in Bogotá had very large numbers of unique plants, and the difference between the plants they report is not explained by geographical factors (the main origin of a human population within different districts in Bogotá) (by 9 geographical zones: *p* = 0.37; by 15 localities: *p* = 0.41) or size (by number of species reported: *p* = 0.22) (Fig. 1). The proportion of unique plants in the markets of Bogotá was generally high and did not show relations to geography or market size (Fig. 2). Even the 11 plant species that were most widespread among all markets did in fact occurred only in relatively few markets (Fig. 3). Not surprisingly, while generally low informant consensus (IC) was highest for introduced species (Table 2).

**Table 2:** Logarithmic informant consensus of Colombia species exceeding the 95%ile (3.0)

Species	LIC
***Sambucus nigra*** **L.**	6.5
***Matricaria chamomilla*** **L.**	6.3
***Ocimum basilicum*** **L.**	5.7
*Lippia alba* (Mill.) N.E. Br. ex Britton & P. Wilson	5.2
*Calendula officinalis* L.	5.1
***Ruta graveolens*** **L.**	5.0
*Aloysia citriodora* Paláu	4.9
***Foeniculum vulgare*** **Mill.**	4.4
*Trichanthera gigantea* (Bonpl.) Nees	4.2
*Juglans neotropica* Diels	4.2
*Persea americana* Mill.	4.0
***Allium sativum*** **L.**	4.0
*Uncaria tomentosa* (Willd.) DC.	3.9
***Valeriana officinalis*** **L.**	3.5
*Ambrosia peruviana* Willd.	3.5
*Equisetum bogotense* Kunth	3.5
*Columnea kalbreyeriana* Mast.	3.4
***Cymbopogon citratus*** **(DC.) Stapf.**	3.3
*Petiveria alliacea* L.	3.2
*Lantana camara* L.	3.2

Introduced species in bold

## Discussion

Overall, species richness encountered in Bogotá was considerably higher than reported in studies in Bolivia [50] but similar to what we encountered in Peru [40–49]. The numbers of species used for each illness was astonishingly high in comparison to the studies in Bolivia and Peru cited above. In contrast to studies in Peru [42, 43, 49], or even Bolivia [50], the species composition and plant use divergence in the markets of Bogotá were also very high. This was in contrast to both our initial hypotheses: While we had expected some discrepancy in plant composition and use in different markets, very much like in Peru [42, 43, 49] and Bolivia [50], the differences between the markets in Bogotá were much larger. Similarly, in contrast to the expectation that larger markets would contain all plant species found in smaller markets, essentially every market in Bogotá was unique. Rather than reflecting shared colonial heritage in plant use, the markets in Bogotá reflected much more the origin of the surrounding population, and each market focused more on the plant resources from the populations’ origin area. This might reflect that the human population of Bogotá, with migrants from all over Colombia, is much more diverse than the populations in the comparison areas (Trujillo-Chiclayo in Peru with mostly coastal population and Andean migrants; La Paz with a mixed population of Quechua and Mestizo origin).

## Conclusions

The present study indicated a very large species and use diversity of medicinal plants in the markets of Bogotá, with profound differences existing even between markets in close proximity. This might be explained by the great differences in the origin of populations in Bogotá, the floristic diversity in their regions of origin, and their very distinct plant use knowledge and preferences that are transferred to the markets through customer demand. Our study clearly indicates that studies in single markets cannot give an in-depth overview on the plant supply and use in large metropolitan areas.

## Abbreviations

FCu: Use reports for a specific use
IC: Informant consensus
ICu: Informant consensus for a specific use
LIC: Logarithmic informant consensus
NSu: Species for a specific use
